# Fine-Mapping of Common Genetic Variants Associated with Colorectal Tumor Risk Identified Potential Functional Variants

**DOI:** 10.1371/journal.pone.0157521

**Published:** 2016-07-05

**Authors:** Mengmeng Du, Shuo Jiao, Stephanie A. Bien, Manish Gala, Goncalo Abecasis, Stephane Bezieau, Hermann Brenner, Katja Butterbach, Bette J. Caan, Christopher S. Carlson, Graham Casey, Jenny Chang-Claude, David V. Conti, Keith R. Curtis, David Duggan, Steven Gallinger, Robert W. Haile, Tabitha A. Harrison, Richard B. Hayes, Michael Hoffmeister, John L. Hopper, Thomas J. Hudson, Mark A. Jenkins, Sébastien Küry, Loic Le Marchand, Suzanne M. Leal, Polly A. Newcomb, Deborah A. Nickerson, John D. Potter, Robert E. Schoen, Fredrick R. Schumacher, Daniela Seminara, Martha L. Slattery, Li Hsu, Andrew T. Chan, Emily White, Sonja I. Berndt, Ulrike Peters

**Affiliations:** 1 Department of Epidemiology and Biostatistics, Memorial Sloan Kettering Cancer Center, New York, NY, United States of America; 2 Public Health Sciences Division, Fred Hutchinson Cancer Research Center, Seattle, WA, United States of America; 3 School of Public Health, University of Washington, Seattle, WA, United States of America; 4 Division of Gastroenterology, Massachusetts General Hospital and Harvard Medical School, Boston, MA, United States of America; 5 Department of Biostatistics, University of Michigan School of Public Health, Ann Arbor, MI, United States of America; 6 Service de Génétique Médicale, CHU Nantes, Nantes, France; 7 Division of Clinical Epidemiology and Aging Research, German Cancer Research Center (DKFZ), Heidelberg, Germany; 8 German Cancer Consortium (DKTK), Heidelberg, Germany; 9 Division of Research, Kaiser Permanente Medical Care Program of Northern California, Oakland, CA, United States of America; 10 Keck School of Medicine, University of Southern California, Los Angeles, CA, United States of America; 11 Division of Cancer Epidemiology, German Cancer Research Center (DKFZ), Heidelberg, Germany; 12 Translational Genomics Research Institute, Phoenix, Arizona, United States of America; 13 Department of Surgery, Mount Sinai Hospital, Toronto, ON, Canada; 14 Division of Epidemiology, Department of Population Health, New York University School of Medicine, New York, NY, United States of America; 15 Melbourne School of Population and Global Health, The University of Melbourne, Melbourne, Victoria, Australia; 16 Ontario Institute for Cancer Research, Toronto, ON, Canada; 17 Departments of Medical Biophysics and Molecular Genetics, University of Toronto, Toronto, ON, Canada; 18 Epidemiology Program, University of Hawaii Cancer Center, Honolulu, HI, United States of America; 19 Department of Molecular and Human Genetics, Baylor College of Medicine, Houston, TX, United States of America; 20 Genome Sciences, University of Washington, Seattle, WA, United States of America; 21 Centre for Public Health Research, Massey University, Wellington, New Zealand; 22 Department of Medicine and Epidemiology, University of Pittsburgh Medical Center, Pittsburgh, PA, United States of America; 23 Division of Cancer Control and Population Sciences, National Cancer Institute, Bethesda, MD, United States of America; 24 Department of Internal Medicine, University of Utah Health Sciences Center, Salt Lake City, UT, United States of America; 25 Channing Division of Network Medicine, Brigham and Women's Hospital and Harvard Medical School, Boston, MA, United States of America; 26 Division of Cancer Epidemiology and Genetics, National Cancer Institute, Bethesda, MD, United States of America; Mayo Clinic Arizona, UNITED STATES

## Abstract

Genome-wide association studies (GWAS) have identified many common single nucleotide polymorphisms (SNPs) associated with colorectal cancer risk. These SNPs may tag correlated variants with biological importance. Fine-mapping around GWAS loci can facilitate detection of functional candidates and additional independent risk variants. We analyzed 11,900 cases and 14,311 controls in the Genetics and Epidemiology of Colorectal Cancer Consortium and the Colon Cancer Family Registry. To fine-map genomic regions containing all known common risk variants, we imputed high-density genetic data from the 1000 Genomes Project. We tested single-variant associations with colorectal tumor risk for all variants spanning genomic regions 250-kb upstream or downstream of 31 GWAS-identified SNPs (index SNPs). We queried the University of California, Santa Cruz Genome Browser to examine evidence for biological function. Index SNPs did not show the strongest association signals with colorectal tumor risk in their respective genomic regions. Bioinformatics analysis of SNPs showing smaller *P*-values in each region revealed 21 functional candidates in 12 loci (5q31.1, 8q24, 11q13.4, 11q23, 12p13.32, 12q24.21, 14q22.2, 15q13, 18q21, 19q13.1, 20p12.3, and 20q13.33). We did not observe evidence of additional independent association signals in GWAS-identified regions. Our results support the utility of integrating data from comprehensive fine-mapping with expanding publicly available genomic databases to help clarify GWAS associations and identify functional candidates that warrant more onerous laboratory follow-up. Such efforts may aid the eventual discovery of disease-causing variant(s).

## Introduction

Genetics play a key role in colorectal cancer (CRC) development [1, 2]; genome-wide association studies (GWAS) have successfully identified many common genetic variants that predict risk [3–19]. Although these variants have modest associations (i.e., per-allele odds ratio less than 1.3), their discovery has reinforced the importance of suspected disease pathways as well as suggested novel ones [20].

An important next step is to characterize the biological importance of these loci. However, single nucleotide polymorphisms (SNPs) identified by GWAS (i.e., index SNPs) are themselves unlikely to be the underlying disease-causing variants; instead, they are expected to tag genomic regions containing correlated SNPs, which may have functional consequences [21, 22]. Laboratory evaluation of all these variants is prohibitively cost- and labor-intensive. Fine-mapping efforts can help inform these experiments by narrowing the size of associated genomic regions likely to contain functional variation [22, 23]. Several recent studies have shown the utility of this approach to refine regions of interest and propose promising functional candidates [14, 17, 24–31].

In addition, some loci may harbor multiple independent risk variants, rather than a single variant. As genomic regions surrounding index SNPs may span more than one linkage disequilibrium block, it is possible these loci harbor additional variants that predict risk independent of the index SNPs. Fine-mapping studies, when conducted within a broader region, can help identify these novel independent risk variants for cancer [14, 31, 32].

In this study of 11,900 colorectal tumor cases and 14,311 controls of European ancestry, we fine-mapped genomic regions harboring 31 known CRC risk variants using both genotyped data and data imputed from the 1000 Genomes Project [33]. This high-density genetic data allowed us to comprehensively examine common (>5%) as well as less common or rare (<5%) genetic variation in these regions. We aimed to narrow the likely region containing the functional variant(s) based on results from association testing, as well as search for novel risk alleles independent of the index SNP. Further, to help inform follow-up laboratory studies, we used a comprehensive bioinformatics-based approach to annotate potential functional candidates.

## Materials and Methods

### Ethics statement

All participants gave written informed consent and this study has been approved by the Fred Hutchinson Cancer Research Center (FHCRC) Institutional Review Board.

### Study population

Details of this study population have been described previously [3, 34] and study-specific descriptions are provided in S1 Text. The study population was derived from studies in the Genetics and Epidemiology of Colorectal Cancer Consortium (GECCO) (13 total: 7 case-control studies nested in prospective cohorts and 6 case-control studies) and the Colon Cancer Family Registry (CCFR) [3, 34]. Study case, control, age, and sex distributions are listed in Table A in S1 Text. We excluded participants of non-European ancestry as determined by principal component analysis [35]. The final study population comprised 11,900 cases (11,074 colorectal cancers, 826 advanced colorectal adenomas) and 14,311 controls.

### Colorectal tumor case definition

Detailed information on case and control definitions is provided in S1 Text. Colorectal cancer cases were defined as colorectal adenocarcinoma confirmed by medical records, pathologic reports, or death certificates. Controls for colorectal cancer cases were population-based or selected from cohort participants who provided a blood sample and had no previous diagnosis of colorectal cancer. Advanced colorectal adenoma cases in the Nurses' Health Study and Health Professionals Follow-Up Study were confirmed by medical records, histopathology, or pathologic reports. Controls for advanced adenoma cases had a negative colonoscopy (except for controls matched to cases with distal adenoma, which either had a negative sigmoidoscopy or colonoscopy exam).

### Genotyping and quality control

Detailed information on genotyping and quality control procedures has been described [3, 34] and are available in S1 Text. Briefly, DNA from blood or buccal samples was genotyped using either Affymetrix (Gene Chip 10K, Mendel) (Affymetrix, Santa Clara, CA) or Illumina arrays (HumanHap550K, 610K, combined 300K and 240K, Human1M, HumanCytoSNP, HumanOmniExpress) (Illumina, Inc., San Diego, CA). Genotyped SNPs were excluded based on call rate (<98%), lack of Hardy-Weinberg Equilibrium in controls (*P*<1x10^-4^), and low minor allele frequency (MAF). All analyses were restricted to samples clustering with the Utah residents of Northern and Western European ancestry, using 1000 Genomes populations as reference, from the Centre d’etude du polymorphisme humain (CEPH) collection (CEU) population in principal component analysis [35].

### Genotype imputation to 1000 Genomes Project

We imputed genotype data to increase the density of genetic variants. As the reference panel we used the haplotypes of 1,092 samples (all populations) from release version 2 of the 1000 Genomes Project Phase I (ftp://ftp-trace.ncbi.nih.gov/1000genomes/ftp/release/20110521) [36]. Combining reference data from all populations helps improve imputation accuracy of low-frequency variants [37]. The target panel comprised genome-wide genotype data obtained using the methods described above. The target panel was phased using Beagle [38], and the phased target panel was imputed to the 1000 Genomes reference panel using Minimac [39]. We used Rsq as the imputation quality measure for imputed SNPs [40]. For imputed SNPs, we required that variants with low MAFs had higher imputation quality: For SNPs with MAF>0.01, we excluded those with Rsq≤0.3; for MAFs of 0.005–0.01, we excluded Rsq<0.5; and for MAF<0.005, we excluded Rsq<0.99.

### Statistical analysis

#### Variant selection

To determine genomic regions for fine-mapping, we identified 31 autosomal SNPs (index SNPs) in 22 loci previously associated with CRC risk in GWAS conducted in European ancestry individuals (Table B in S1 Text) [3–18]. Through fine-mapping, we aimed to (1) refine the location of potential functional candidate(s) tagged by these index SNPs and (2) identify novel or independent signals near these loci, the latter being hypothesis-generating. We thus defined a broad genomic region of interest as that spanning 250-kb upstream and downstream of each index SNP and evaluated all variants in this 500-kb interval.

#### Association testing

All statistical analyses were conducted centrally at the GECCO coordinating center on individual-level data to ensure a consistent analytical approach. Unless otherwise indicated, as appropriate, we adjusted for age at the reference time, sex, study center, smoking status (PHS only), batch effects (ASTERISK only: upon quality control there were slight variations in genotyping quality across batches, which were not observed in other studies), and the first three principal components from EIGENSTRAT [35] to account for population substructure.

For each study, we estimated the association between individual variants and colorectal tumor risk by calculating odds ratios (ORs) and 95% confidence intervals (CIs) using unconditional logistic regression assuming log-additive genetic effects. Each genotyped SNP was coded as 0, 1 or 2 copies of the variant allele. For imputed SNPs, we used the expected number of copies of the variant allele (the dosage), which gives unbiased effect estimates [41]. To combine study-specific estimates across studies, we obtained summary estimates using inverse-variance weighted fixed-effects meta-analysis. Colorectal cancer cases and their controls were analyzed separately from advanced adenoma cases and their controls before meta-analysis. We calculated heterogeneity *P*-values using Cochran's Q statistics [42]. Quantile-quantile (Q-Q) plots were used to assess whether the distribution of *P*-values was consistent with the null distribution (except for the extreme tail). In each region, we searched for additional independent association signals by testing each of the other variants conditioned on the top SNP in that region (i.e., 2 variants included in each model); variants are expected to be less correlated after conditioning on the top SNP. When testing for additional independent signals, we determined the *P*-value threshold for statistical significance by using the number of SNPs in each 500-kb region as the Bonferroni correction factor (α-level for a region = 0.05/# SNPs in that region). We used this approach to correct for multiple testing while also accounting for the knowledge that genetic variation in these regions is known to influence predisposition to CRC.

We used R (Version 2.15.1, R Foundation for Statistical Computing, Vienna, Austria) to conduct the statistical analysis, and LocusZoom [43] to visualize results. To determine the minimum detectable effect estimates in the present analysis, we estimated statistical power using Quanto Version 1.2.4 (http://hydra.usc.edu/gxe/).

### Functional annotation using bioinformatics

Detailed information on functional annotation and various databases is provided in S1 Text. In brief, compared with variants that are either non-functional or not in linkage disequilibrium with the underlying functional variant(s), colorectal tumor association signals are expected to be strongest (show the smallest *P*-value) for the functional variant(s), or variants in high linkage disequilibrium with the functional variant(s). Thus we selected the following for bioinformatics follow-up: 1) the variant showing the strongest evidence for association (smallest *P*-value) in each region (i.e., top SNP), 2) the index SNP in each region, 3) among the top 10 variants with the smallest *P*-values in each region, those that were correlated (*r*^*2*^>0.5 in 1000 Genomes European populations) with the index SNP, and 4) any SNP completely correlated (*r*^*2*^ = 1 in 1000 Genomes European populations) with any SNP listed in parts 1–3. In addition, after performing conditional analyses that simultaneously included the index SNP(s) in multivariable models, we annotated SNPs showing *P* ≤ 5E-05 and any SNPs completely correlated with these.

We annotated the potential function of variants in coding regions using PolyPhen-2 [44]. For variants in non-coding regions, we used HaploReg [45, 46] and the University of California, Santa Cruz (UCSC) Genome Browser [47] to align each SNP to the reference genome and annotate them with multiple datasets generated from the Encyclopedia of DNA Elements (ENCODE) Project [48, 49] or the NIH Roadmap program on Epigenomics [50] as detailed in S1 Text. Annotation using these databases assumes that the disease-causing variant(s) affects disease by altering gene transcription through multiple regulatory mechanisms [48, 49]. Such mechanisms include indicators for regions that may influence transcriptional regulation of target genes, such as chromatin accessibility (open chromatin), histone modification, binding of regulatory proteins, and alteration of regulatory motifs [45, 51, 52]. Conservation across vertebrates can provide further evidence of biologically important regions [53, 54]. To identify variants showing any of these indicators of functional importance, we first queried HaploReg [45, 46], which provides an overview of available annotations. We further interrogated variants with any functional evidence using the UCSC Genome Browser [47] to examine signal enrichment in regions harboring these variants, which helps correct for false positive signals for each assay (https://sites.google.com/site/anshulkundaje/projects/idr). Specifically, we examined whether variants were located in functionally important regions using the following datasets compiled by HaploReg [46] or UCSC Genome Browser [47]: DNAse I hypersensitivity data in ENCODE cell lines, including two for CRC (HCT-116 and Caco-2), to assess *open chromatin structure*; ChIP-seq data in ENCODE cell lines as well as Roadmap data in normal colon and rectal tissues for *histone enhancer or promoter modifications*; ChIP-seq data in ENCODE cell lines to determine regions that *bound to important regulatory proteins* (e.g., promoters, enhancers, silencers, and insulators); change in log-odds score based on position weight matrices [45] to predict whether a sequence harboring either the reference or alternate allele would exhibit *altered binding affinities* for regulatory proteins; and PhastCons scores [53, 54] to predict genomic regions *conserved across vertebrates*. As intergenic variants often regulate the closest downstream gene [48, 49], we predicted the gene regulated by each variant based on proximity of each variant to a gene as well as the orientation (3’ or 5’) relative to the nearest end of the gene [45]. Recognizing that cis-regulatory elements can also skip the closest gene, in exploratory analyses we integrated expression quantitative trail locus (eQTL) analysis to identify other potential tissue-specific target genes from the Genotype-Tissue Expression (GTEx) database [46], GEUVADIS [55], and other recent studies [56–60] using HaploReg and the GTEx Portal. Further, we evaluated variants in potential splice sites using Genie [61].

The relative strength of functional candidates was determined based on the accumulation of evidence from each of these datasets. *A priori*, we defined a score to summarize the amount of functional evidence for each variant using the following algorithm: showed (+1) histone modification, (+1) open chromatin, (+1) protein binding, (+1) protein binding in the presence of open chromatin or histone modification, (+1) different patterns of histone modification in cancerous vs. noncancerous cell lines/ tissues, (+1) regulatory evidence in a CRC cell line (e.g. Caco-2 or HCT-116) or normal colon/rectal tissue, (+0.5) altered binding motif, and (+0.5) a conserved region across vertebrates. Thus, variants were assigned a maximum score of 7. Although not observed in our data, any variant in a coding region predicted by PolyPhen to be “possibly damaging” or “probably damaging” would have been assigned a score of 8 or 9, respectively. Variants in coding regions predicted by PolyPhen to be “benign” or “unknown” were scored as a non-coding regulatory variant, as DNA sequences can act as coding exons in one tissue and enhancers of nearby gene(s) in another [62]. Caution should be exercised when interpreting these scores as there is a degree of uncertainty when relating annotation data to SNP function. These data are based on transformed cell lines or tissues instead of living organisms, and regulatory mechanisms may vary temporally as well as across different types of cells or tissues. However, bioinformatics analysis is primarily useful for prioritizing a large number of variants for more onerous laboratory follow-up; to this end, we used these scores to create 3 categories that ranked the strength of functional evidence for each variant: score of 3–3.5 =“weak”, 4–4.5 =“moderate”, and ≥5 =“strong”.

## Results

The mean age of the 26,211 participants was 64.2 years, ranging from 19 to 99 years (Table A in S1 Text). Two studies (HPFS, PHS) comprised only males, and 3 studies (NHS, PMH, WHI) only females. The proportion of females in the remaining studies ranged from 30.8% to 52.0%.

For the 31 previously reported CRC-related variants (index SNPs), 17 showed *P*-values ≤ 0.001, 22 showed *P*-values ≤ 0.01, and 27 showed *P*-values ≤ 0.05 (Table B in S1 Text). Further, ORs for 30 of 31 SNPs showed directions consistent with previous findings.

**Fig 1:**
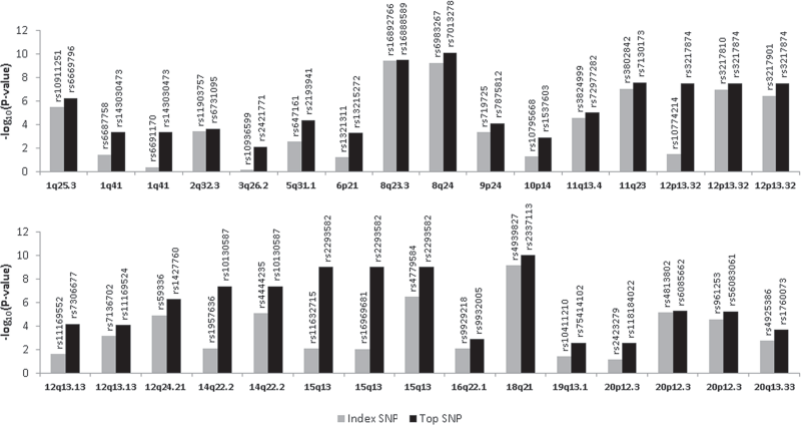
Comparison of *P*-values for GWAS-identified variants (index SNPs) vs. variants with the smallest *P*-values (top SNPs) in 31 regions. The height of each bar reflects the–log10 *P*-value of each SNP in our study population. A grey bar indicates the index SNP, and a black bar indicates the top SNP.

Across the 31 genomic regions encompassing index SNPs, there were on average 1,807 SNPs per 500-kb region, ranging from 967 to 2,364 SNPs per region. SNPs with the strongest evidence of CRC-associations may more likely be functional or strong proxies for functional candidates. To help refine regions harboring functional candidates, we identified the SNP showing the smallest *P*-value in each region (i.e., top SNP) (Table 1). The initial index SNP did not show the strongest association signal in any genomic region (Fig 1). For loci that harbored more than 1 index SNP, the regions encompassing each index SNP sometimes overlapped, yielding regions in which the top SNP was the same (e.g., at 1q41, the top SNP rs143030473 showed the smallest *P*-value in 2 regions, defined by index SNPs rs6687758 and rs6691170). This was observed in 1q41, 12p13.32, 14q22.2, and 15q13. Thus for the 31 regions studied there were 25 unique top SNPs (note 12p13.32 and 15q13 each contained 3 index SNPs); of these, 20 had an association with *P*-values ≤ 0.001 and all 25 showed *P*-values ≤ 0.01. For these 25 variants, the top SNP was correlated with an index SNP in European populations at *r*^*2*^ ≥ 0.8 for 8 SNPs, 0.6 ≤ *r*^*2*^ < 0.8 for 6 SNPs, 0.4 ≤ *r*^*2*^ < 0.6 for 4 SNPs, 0.2 ≤ *r*^*2*^ < 0.4 for 1 SNP, and *r*^*2*^ < 0.2 for the remaining 6 SNPs.

**Table 1:** Association results for variants showing the smallest *P*-values (top SNPs) in 31 regions surrounding previous GWAS-identified variants (index SNPs).

Locus	Index SNP	Level ^a^	# SNPs	Top SNP	Level ^a^	Position ^b^	Genetic	*r*^2^ with	Top SNP results				
			in				region	index	Ref/	Ref	OR (95% CI) ^d^^,^^e^	*P*	*P*-het
			region					SNP ^c^	other	allele			
									allele	freq			
1q25.3	rs10911251	***	1886	rs6669796	***	183082825	*LAMC1*	0.87	G/C	0.56	1.11 (1.07, 1.16)	5.8E-07	6.0E-01
1q41	rs6687758	*	1885	rs143030473	***	222161943	*DUSP10/CICP13*	<0.2	C/T	0.99	1.97 (1.35, 2.87)	4.5E-04	9.2E-01
	rs6691170		2096	rs143030473	***	222161943	*DUSP10/CICP13*	<0.2	C/T	0.99	1.97 (1.35, 2.87)	4.5E-04	9.2E-01
2q32.3	rs11903757	***	1536	rs6731095	***	192589442	*NABP1/SDPR*	1.00	A/G	0.84	0.89 (0.84, 0.95)	2.3E-04	1.1E-01
3q26.2	rs10936599		1651	rs2421771	**	169411370	*MECOM/MYNN*	<0.2	C/T	0.96	1.21 (1.05, 1.40)	7.5E-03	9.2E-01
5q31.1	rs647161	**	1499	rs2193941	***	134469594	*PITX1/ H2AFY*	0.57	G/A	0.72	1.09 (1.05, 1.14)	4.0E-05	9.2E-01
6p21	rs1321311		2364	rs13215272	***	36589502	*SRSF3/CDKN1A*	<0.2	C/T	0.73	1.10 (1.04, 1.15)	5.3E-04	8.0E-02
8q23.3	rs16892766	***	1432	rs16888589	***	117635602	*TRPS1/EIF3H*	0.92	A/G	0.91	0.80 (0.75, 0.86)	3.3E-10	7.1E-01
8q24	rs6983267	***	2257	rs7013278	***	128414892	*SRRM1P1/POU5F1B/MYC*	0.40	C/T	0.66	0.88 (0.85, 0.92)	7.8E-11	7.4E-01
9p24	rs719725	***	1907	rs7875812	***	6364533	*TPD52L3/UHRF2*	1.00	A/T	0.63	1.09 (1.04, 1.13)	7.9E-05	7.4E-01
10p14	rs10795668	*	2363	rs1537603	**	8734295	*KRT8P16/TCEB1P3*	0.45	C/T	0.50	1.06 (1.02, 1.10)	1.2E-03	7.9E-01
11q13.4	rs3824999	***	1788	rs72977282	***	74300441	*LIPT2/POLD3*	0.59	T/A	0.59	0.92 (0.88, 0.95)	9.8E-06	8.1E-01
11q23	rs3802842	***	1830	rs7130173	***	111154072	*C11orf93*	0.95	C/A	0.73	0.89 (0.85, 0.93)	2.7E-08	3.8E-01
12p13.32	rs10774214	*	1656	rs3217874	***	4400808	*CCND2*	<0.2	C/T	0.58	0.89 (0.85, 0.93)	3.1E-08	7.8E-01
	rs3217810	***	1571	rs3217874	***	4400808	*CCND2*	<0.2	C/T	0.58	0.89 (0.85, 0.93)	3.1E-08	7.8E-01
	rs3217901	***	1539	rs3217874	***	4400808	*CCND2*	0.65	C/T	0.58	0.89 (0.85, 0.93)	3.1E-08	7.8E-01
12q13.13	rs11169552	*	1310	rs7306677	***	51205763	*ATF1*	<0.2	C/T	0.62	0.92 (0.89, 0.96)	6.3E-05	3.4E-01
	rs7136702	***	967	rs11169524	***	51089734	*DIP2B*	0.67	T/A	0.67	0.92 (0.88, 0.96)	8.5E-05	5.3E-01
12q24.21	rs59336	***	2072	rs1427760	***	115100714	*TBX5/TBX3*	0.71	T/C	0.50	0.90 (0.87, 0.94)	5.0E-07	2.5E-01
14q22.2	rs1957636	***	1613	rs10130587	***	54419110	*BMP4*	<0.2	G/C	0.63	0.89 (0.85, 0.93)	4.1E-08	9.0E-01
	rs4444235	***	1659	rs10130587	***	54419110	*BMP4*	0.67	G/C	0.63	0.89 (0.85, 0.93)	4.1E-08	9.0E-01
15q13	rs11632715	**	1735	rs2293582	***	33010412	*GREM1*	0.23	G/A	0.80	0.86 (0.82, 0.91)	1.0E-09	3.8E-01
	rs16969681	**	1692	rs2293582	***	33010412	*GREM1*	0.22	G/A	0.80	0.86 (0.82, 0.91)	1.0E-09	3.8E-01
	rs4779584	***	1701	rs2293582	***	33010412	*GREM1*	0.71	G/A	0.80	0.86 (0.82, 0.91)	1.0E-09	3.8E-01
16q22.1	rs9929218	**	1867	rs9932005	**	68822019	*CDH1*	0.63	C/T	0.76	1.08 (1.03, 1.13)	1.2E-03	6.6E-01
18q21	rs4939827	***	1931	rs2337113	***	46452327	*SMAD7*	0.91	A/G	0.55	1.13 (1.09, 1.18)	1.0E-10	2.5E-01
19q13.1	rs10411210	*	2307	rs75414102	**	33302424	*TDRD12/SLC7A9*	<0.2	G/A	0.99	0.51 (0.33, 0.79)	2.6E-03	9.1E-01
20p12.3	rs2423279		1901	rs118184022	**	7719449	*BMP2/HAO1*	<0.2	C/T	0.98	0.58 (0.41, 0.83)	2.8E-03	9.4E-01
	rs4813802	***	1825	rs6085662	***	6698372	*FERMT1/BMP2*	1.00	G/C	0.65	0.91 (0.87, 0.95)	5.1E-06	3.0E-01
	rs961253	***	1990	rs56083061	***	6430696	*FERMT1/BMP2*	0.28	G/A	0.79	0.90 (0.85, 0.94)	5.6E-06	7.6E-01
20q13.33	rs4925386	**	2173	rs1760073	***	60926106	*LAMA5*	0.81	G/A	0.67	1.08 (1.04, 1.12)	1.9E-04	8.8E-02

Abbreviations: SNP, single nucleotide polymorphism; Ref, reference; Freq, frequency; OR, odds ratio; CI, confidence interval; *P*-het, *P* value for test of heterogeneity across studies

^a^Level of statistical evidence:

*SNP with *P* ≤ 0.05

**SNP with *P ≤* 0.01

***SNP with *P*≤0.001. *P*-values are not adjusted for multiple testing as these data are intended to help refine previously identified CRC-related regions and highlight sets of variants warranting bioinformatics-based follow-up.

^b^Based on NCBI build 37 data.

^c^Based on 1000 Genomes Project data in European populations.

^d^Adjusted for age in years, sex, first 3 principal components, study center, batch (ASTERISK only), and smoking (PHS only).

^e^Estimate calculated using the log-additive genetic model for each additional reference allele.

Variants carried forward for functional annotation spanned a median interval of 32-kb. We scored 21 variants in 12 loci as having “strong” functional evidence (Table 2, additional details in S1 Table). At 4 loci (8q24, 11q13.4, 19q13.1, 20p12.3) the index SNP was among the SNPs with the highest functional scores. All 21 candidates were located in regions that were non-coding (15 intronic and 6 intergenic) with open chromatin structure (i.e., accessible to regulatory factors). Twenty of 21 candidates (all except for 18q21/rs34007497) bound to multiple transcription factors. Fifteen variants were predicted to disrupt transcription factor binding. Several candidates showed different patterns of histone enhancer or promoter marks when comparing cancer cells vs. normal cells or tissues. Only 3 variants (8q24/rs6983267, 18q21/rs4939567, 20p12.3/rs4813802) were located in an evolutionarily conserved region, suggesting that most of the predicted regulatory regions may be dynamic through evolution.

**Table 2:** SNPs showing strong evidence for functional importance based on bioinformatics.

**Locus**	Index SNP	Strong	Mean	*r*^*2*^ with	Predicted	Genomic	# Cell lines	Show histone regulatory marks ^e^			Proteins	Altered binding	Evidence
		functional	imput-	index	regulated	location of	showing	# Cancer/	# Normal	Normal	bound ^e^	motifs ^g^	for
		candidate	ation	SNP ^b^	gene ^c^	functional	open	progenitor	cell lines	colorectal			conserved
			*r*^*2*^ ^a^			candidate	chromatin ^d^	cell lines		tissues ^f^			region ^h^
5q31.1	rs647161	rs1366111	0.89	0.57	*PITX1*	intronic	2	2	—	1, 2	POL2,EGR1	Zfx	0
8q24	rs6983267	rs6983267	0.95	index	*MYC*	intergenic	1 CRC line	1	2	2	TCF4 (CRC line),	AP-1, Sox, TCF4	1
											P300, FOXA1,		
											RXRA, SP1		
11q13.4	rs3824999	rs3824999	0.99	index	*POLD3*	intronic	4	—	2	4	JUND	AP-3, Evi-1, Mef2	0
11q23	rs3802842	rs7130173	0.95	0.93	*Unknown*	intronic	31 including	—	—	2, 3	RAD21, SMC3,	GSP1, SRF	0
							1 CRC line				YY1, CTCF		
12p13.32	rs3217901	rs3217827	0.85	0.61	*CCND2*	intronic	1	—	—	3	RAD21, CTCF	—	0
12q24.21	rs59336	rs71807	0.89	0.86	*TBX3*	intergenic	1	2	4	1, 3	BAF155, HAE2F1,	—	0
											CTCF		
		rs484443	0.91	0.90	*TBX3*	intronic	8	1	2	2, 4	P300,USF1	RREB-1, Rad21	0
14q22.2	rs4444235	rs2071047	1.00	0.75	*BMP4*	intronic	9	3	3	1–4	TCF4 (CRC line)	BCL, HNF4, RXRA	0
		rs10130587	0.74	0.67	*BMP4*	intronic	2	3	3	1–4	GATA3	5 motifs	0
		rs35107139	0.82	0.64	*BMP4*	intronic	2	3	3	1, 3, 4	GATA3	12 motifs	0
15q13	rs4779584	rs2293582	0.94	0.71	*GREM1*	intronic	4	3	5	1–4	POL2	—	0
		rs2293581	0.97	0.66	*GREM1*	intronic	36	4	5	1–4	SUZ12	—	0
		rs1406389	0.97	0.66	*GREM1*	intergenic	12	3	4	1, 2, 4	SUZ12	Irf, SIX5	0
18q21	rs4939827	rs11874392	0.93	1.00	*SMAD7*	intronic	4	3	1	1, 2, 4	11 proteins	—	1
		rs4939567	0.91	1.00	*SMAD7*	intronic	1	4	3	1–4	MAFK	RREB-1, VDR_2	1
		rs34007497	0.91	0.57	*SMAD7*	intronic	22	2	4	1–4	CTCF,STAT1	4 motifs	0
19q13.1	rs10411210	rs10411210	0.94	index	*RHPN2*	intronic	48 including	2	1	1, 2	17 proteins	Mef2, STAT	1
							1 CRC line						
20p12.3	rs961253	rs966817	1.00	0.87	*BMP2*	intergenic	18	—	1	—	CJUN	Evi-1, FAC1, GLI	1
	rs4813802	rs4813802	0.86	index	*BMP2*	intergenic	10	2	2	2	6 proteins	—	1
		rs6085661	0.89	0.76	*BMP2*	intergenic	19	1	—	2	7 proteins	BCL, NRSF, VDR	1
20q13.33	rs4925386	rs1741634	0.96	0.80	*LAMA5*	intronic	7	—	2	1, 2	GR	EWSR1-FLI1	0

^a^Estimated value of the squared correlation between imputed genotypes and true (unobserved) genotypes for the strong functional candidate, averaged across studies.

^b^Based on data from 1000 Genomes Pilot 1 CEU.

^c^Based on HaploReg data using proximity of each variant to a gene as well as the orientation (3’ or 5’) relative to the nearest end of the gene.

^d^Based on data from DNAse I hypersensitivity assays.

^e^ENCODE ChIP-seq assay.

^f^Based on data from ChIP-seq assays in the Roadmap Epigenomics Project in the following normal (non-cancerous) tissues: (1) colon mucosa, (2) rectal mucosa, (3) colon smooth muscle, and (4) rectal smooth muscle.

^g^Position weight matrix score between SNP alleles confers a change in log-odds (LOD) score > 5.

^h^Based on PhastCons scores: 1 = strong evidence of conserved element; 0 = no evidence of conserved element.

In each region, after conditioning on the top SNP and accounting for the number of tests, we did not observe any statistically significant SNPs.

## Discussion

In this large study population of over 26,000 participants of European ancestry, we used high-density genetic data imputed from the 1000 Genomes Project to comprehensively fine-map genomic regions harboring 31 GWAS-identified CRC risk variants. In association tests, the index SNP did not show the smallest *P*-value in any genomic region. Using bioinformatics-based annotation to follow-up variants with the strongest association signals, we showed strong evidence for 21 functional candidates in 12 CRC-related loci. We observed limited evidence of additional independent CRC association signals within GWAS-identified regions.

Although the index SNP did not show the smallest *P*-value in any genomic region, all functional candidates were correlated with the index SNP (*r*^*2*^ of at least 0.57). Interestingly, however, the index SNP was a strong functional candidate in only 4 of the 12 loci harboring a strong functional candidate. Combined, these data from our association testing and functional annotation support the hypothesis that most GWAS-identified index SNPs are not the underlying functional variant, but may instead act as proxies of correlated variants with biological importance.

Eight previous studies have fine-mapped a limited number of GWAS-identified CRC loci in individuals of European ancestry [14, 17, 24–29]; these studies have reported 34 candidate SNPs showing functional evidence (summarized in Table C in S1 Text). In addition, 2 recent studies have comprehensively fine-mapped known CRC loci: Whiffin et al. [31] identified 4 additional candidates in 1q41, 15q13, 18q21, and 20q13.33 in European ancestry individuals (5,626 cases; 7,817 controls); Wang et al. [30] identified 1 additional candidate in 1q41 in African Americans (1,894 cases; 4,703 controls). Of these 39 reported candidates in 11 loci, 36 passed genotyping quality control in our study. In the present analysis, 16 of these SNPs had *P*-values ≤ 0.001, 5 had *P*-values >0.001 and ≤ 0.01, and 7 had *P*-values >0.01 and ≤ 0.05. Similar to our findings, only 5 of 39 previously reported functional candidates were GWAS-identified SNPs. We observed 3 exonic candidates out of an expanded list of 51 variants showing “weak”, “moderate”, or “strong” functional evidence (see S1 Table for an expanded list of functional candidates); similarly, only 2 previously reported candidates (rs706793, rs28626308) were in coding regions [24, 25]—highlighting the importance of non-coding effects on CRC [63].

To identify potential variants for laboratory follow-up, we compared all previously reported candidates (in 11 loci) with variants in the present analysis that showed “moderate” or “strong” functional evidence (Table C in S1 Text). In 5 loci (8q23.3, 8q24, 15q13, 18q21, and 19q13.1) our data confirmed previously reported functional candidates [25, 27, 29, 31]. In addition, in 11q23 and 16q22.1 we observed candidate(s) that highly correlated and were within 5-kb of a previously reported candidate variant [25, 28]. Fine-mapping can be limited in distinguishing which of several highly correlated SNPs located very close together is the true causal variant. Our candidates in these 2 loci, 11q23/rs7130173 and 16q22.1/rs9929218, showed stronger functional evidence compared with reported candidates, which either were not selected for functional annotation or showed less than “weak” functional evidence (scored less than 3). These data show that to avoid missing functional variation, laboratory studies should follow-up not just the strongest candidates, but also variants showing any evidence of biological importance that are very close and highly correlated. Our data did not show functional evidence for reported candidates in the remaining 4 loci. In 1q41 we did not identify a functional candidate; in 12q13.13 and 14q22.2 we predicted functional candidates that were >150-kb from those previously reported [14, 24]; and in 20q13.33 our candidates were >5-kb away and did not show high correlation with those previously reported (*r*^*2*^ = 0.59–0.60) [31]. In 3 of these loci (1q41, 12q13.13, 14q22.2) only 1 of 7 previously reported candidates showed *P*<0.05 in our study population, suggesting they may be false positives. In 20q13.33 the reported candidates, rs1741640 and rs2236202, were not selected for functional annotation in our study based on their *P*-values relative to other variants in the region. Taken together, these data support the utility of fine-mapping to reveal potential functional variation, but also highlight that these studies only serve as an initial step toward determining the underlying causal variant(s) that lead to disease. Results from bioinformatics-based annotation depend on various factors (e.g., queried variants, queried databases, choice of cell lines and tissues, uncertainty in interpreting data from qualitative assays, among others), which vary between studies. It is likely these differences in methodology and interpretation when annotating variants account in part for inconsistencies in results. Consequently, replication of fine-mapping findings is useful, and only targeted functional studies can provide more definitive evidence of SNP function [22, 23].

In our study, for instance, the 500-kb region containing rs6983267 (8q24) harbored 2,257 SNPs. Based on association testing, we narrowed this region to a 13-kb interval that included 7 correlated SNPs showing stronger association signals (Figure A panel A in S1 Text). After bioinformatics analysis, the best functional candidate was the index SNP rs6983267, which was predicted to alter the binding of TCF4 transcription factor. Consistent with this, Tuupanen et al. [27] showed *in vitro* and *in vivo* that rs6983267 resulted in differential TCF4 binding, which may result in enhanced responsiveness to Wnt signaling and a subsequent increase in risk. Further, several other laboratory experiments support the biological importance of this variant in CRC [64–66]. Similarly, the 500-kb region containing rs3802842 (11q23) harbored 1,830 SNPs. Association tests narrowed this region to an 18-kb interval that included 9 correlated SNPs for which we performed bioinformatics follow-up (Figure A panel B in S1 Text). Among these, rs7130173 showed strong regulatory evidence in our study. Consistent with these findings, Biancolella et al. [67] recently showed that the risk allele of rs7130173 reduced enhancer activity and resulted in reduced transcription factor binding affinity in CRC cells. A combination of fine-mapping and laboratory functional follow-up has also shown similar successes for other cancers and chronic diseases [23, 68, 69]. Taken together, these data suggest that by combining association testing and bioinformatics analysis, fine-mapping can reduce the size of relevant genomic regions and successfully prioritize candidates for molecular characterization, which greatly reduces the cost, time, and labor associated with testing a large number of variants in the laboratory.

In addition to confirming previous candidates, we suggest several novel candidates with strong functional evidence. These, located in 4 loci with previously reported functional candidates (12q13.13, 14q22.2, 15q13, 20q13.33) and 5 loci without any previously reported candidates (5q31.1, 11q13.4, 12p13.32, 12q24.21, 20p12.3), implicated genes expected to be involved in CRC development as well as those that were unexpected. For instance, duplication in the *GREM1* (gremlin 1) promoter has been linked to hereditary mixed polyposis syndrome [70], suggesting it is a candidate gene for colorectal tumorigenesis. In our analysis rs2293582, an intronic SNP in *GREM1* (15q13), showed the smallest *P*-value in this region and was among our best functional candidates. The region containing rs2293582 exhibited open chromatin and bound RNA Polymerase 2 *in vivo* (ENCODE tracks shown in Figure B in S1 Text). This region also showed strong promoter marks in colon cancer cell lines, but greatly reduced signals in normal colon and rectal tissues. These data suggest rs2293582 warrants experimental follow-up, along with two highly correlated variants within 1-kb, rs2293581 (*r*^*2*^ = 0.94) and a previously reported candidate rs1406389 (*r*^*2*^ = 0.94) [31], located in regions showing histone marks, open chromatin, and binding to the repressive transcription factor SUZ12 [71]. Fine-mapping can also help identify functional candidates that implicate unexpected genes for further functional study. *LAMA5* (laminin, alpha 5), for instance, is involved in maintaining the extracellular matrix [72], which may not be expected to predict cancer risk. The SNP showing the smallest *P*-value in the 20q13.33/*LAMA5* region, rs1760073, was completely correlated (*r*^*2*^ = 1) with the best functional candidate, rs1741634, which was located in an intron of *LAMA5*. The region containing rs1741634 exhibited open chromatin, bound the glucocorticoid receptor transcription factor, which has been implicated in cancer [73], and interestingly, was located in a region showing different enhancer marks in CRC cell lines vs. normal colon and rectal tissues (Figure C in S1 Text). In addition, Whiffin et al. [31] recently reported other functional candidates in this region. Thus, although unexpected, these data, along with those from GWAS showing associations with a variant in another laminin gene, *LAMC1* (laminin, gamma 1) [3, 19], support the role of laminin proteins in colorectal carcinogenesis.

Particular strengths of this study included the large study population, high-density genetic data, as well as systematic approach to fine-mapping all GWAS-identified CRC risk variants; however, limitations should be noted. As we aimed to comprehensively investigate both common and less common genetic variation, we examined directly genotyped SNPs as well as SNPs imputed from the 1000 Genomes Project. Imputed genotypes can be called with varying accuracy, and we accounted for this using the genotype dosage, which have been shown to yield unbiased estimates [41]. However, lower imputation accuracy may attenuate the estimated significance of association signals [74, 75], and thus relative *P*-values for individual variants may not necessarily correspond to their functional importance. Accordingly, rather than only assessing the SNP showing the smallest *P*-value in each region we identified a set of SNPs showing stronger association signals for bioinformatics analysis, which enabled us to reduce considerably the number of potential functional SNPs per region and still be able to identify promising functional candidates. Even within our large study, limited statistical power may have accounted for the absence of less common independent association signals at known CRC susceptibility loci, particularly for regions where the initial GWAS showed weak effects. For common genetic variants (allele frequency = 20%), the present analysis had 80% power to detect a per-allele OR of 1.12; for less common variants (allele frequency = 1%), there was 80% power to detect a per-allele OR of 1.51 (Figure D in S1 Text). These estimates suggest that although larger populations are likely needed to detect weaker associations with less common variants, our data provided sufficient statistical power to detect less common SNPs with larger effect sizes.

In this large population, we comprehensively fine-mapped known common variants that predict CRC risk. We refined genomic regions harboring risk variants and proposed novel functional candidates, as well as confirmed several regions previously reported to contain functional variation. These findings support the utility of a systematic fine-mapping approach that integrates information from expanding publicly available databases to help refine regions surrounding GWAS-identified risk variants and identify a limited number of functional candidates. These insights may help establish a framework for follow-up laboratory studies, which are necessary to yield definitive evidence of functional SNPs that drive common genetic predisposition to CRC.

## Supporting Information

S1 Table Expanded list of SNPs with weak, moderate, or strong evidence of biological function based on bioinformatics (including those in Table 2). (XLSX)

S1 Text Supplementary materials. Describes in detail the study population and case/control definition; genotyping and quality control; as well as functional annotation using bioinformatics. Also includes Supplementary Tables A-C and Supplementary Figures A-D.
(DOCX)
