# Supplementary material for: Fine-Mapping of Common Genetic Variants Associated with Colorectal Tumor Risk Identified Potential Functional Variants
Source: PLoS One. 2016 Jul 5;11(7):e0157521. doi: 10.1371/journal.pone.0157521 (PMC4933364; doi:10.1371/journal.pone.0157521)
Supplement: S1 Text — Describes in detail the study population and case/control definition; genotyping and quality control; as well as functional annotation using bioinformatics. Also includes Supplementary Tables A-C and Supplementary Figures A-D. (DOCX) [file pone.0157521.s002.docx]

**S1 TEXT. Supplementary materials**

**Fine-Mapping of Common Genetic Variants Associated with Colorectal Tumor Risk Identified Potential Functional Variants**

**Authors:**

Mengmeng Du ♠, Shuo Jiao ♠, Stephanie A. Rosse, Manish Gala, Goncalo Abecasis, Stephane Bezieau, Hermann Brenner, Katja Butterbach, Bette J. Caan, Christopher S. Carlson, Graham Casey, Jenny Chang-Claude, David V. Conti, Keith R. Curtis, David Duggan, Steven Gallinger, Robert W. Haile, Tabitha A. Harrison, Richard B. Hayes, Michael Hoffmeister, John L. Hopper, Thomas J. Hudson, Mark A. Jenkins, Sébastien Küry, Loic Le Marchand, Suzanne M. Leal, Polly A. Newcomb, Deborah A. Nickerson, John D. Potter, Robert E. Schoen, Fredrick R. Schumacher, Daniela Seminara, Martha L. Slattery, Li Hsu, Andrew T. Chan, Emily White, Sonja I. Berndt, Ulrike Peters

♠ Authors contributed equally

**Description of study population and case/control definition**

The present analysis included individuals of European ancestry who were selected as cases or controls in a recently published GWAS of CRC [[1](#_ENREF_1)]. The study population comprised the Colon Cancer Family Registry (CCFR) and the Genetics and Epidemiology of Colorectal Cancer Consortium (GECCO). GECCO included the following nested case-control studies in prospective US cohorts: Health Professionals Follow-up Study (HPFS); Multiethnic Cohort Study (MEC); Nurses’ Health Study (NHS); Physician’s Health Study (PHS); Prostate, Lung, Colorectal and Ovarian Cancer Screening Trial (PLCO); VITamins And Lifestyle (VITAL); Woman’s Health Initiative (WHI); as well as the following case-control studies from the US, Canada, and Europe: French Association Study Evaluating RISK for sporadic colorectal cancer (ASTERISK); Colorectal Cancer Studies 2&3 (Colo2&3); Darmkrebs: Chancen der Verhütung durch Screening (DACHS); Diet, Activity and Lifestyle Survey (DALS); Ontario Familial Colorectal Cancer Registry (OFCCR); and Postmenopausal Hormone Study-Colon Cancer Family Registry (PMH-CCFR). In the following we describe each study population and the definitions of cases and controls used in the present analysis. For information on sample sizes as well as distributions of case, control, age, and sex across the 14 studies please see Supplementary Table A.

**French Association Study Evaluating RISK for sporadic colorectal cancer (ASTERISK)** [[2](#_ENREF_2)]. Participants were recruited from the Pays de la Loire region in France between December 2002 and March 2006. Eligibility criteria for cases included being of Caucasian origin, being greater than or 40 years of age at diagnosis, and having no family history of colorectal cancer or polyps. Cases were patients with first primary colorectal cancer diagnosed in one of the six public hospitals and five clinics located in the Pays de la Loire region which participated in the study. Cases were confirmed based on medical and pathology reports. Controls were recruited at two Health Examination Centers of the Pays de la Loire region, and the recruitment of controls greater than or 70 years was completed in the departments of internal medicine and hepatogastroenterology of the University Hospital Center of Nantes, located in the same region. Controls were eligible to participate if they were Caucasian, aged greater than or 40 years, and had no family history of colorectal cancer or polyps. In the presence of the physician, each participant filled out a standardized questionnaire on family information, medical history, lifestyle, and dietary intake. Cases and controls provided a blood sample.

**Colon Cancer Family Registry (CCFR)**. The CCFR is an NCI-supported consortium consisting of six centers dedicated to the establishment of a comprehensive collaborative infrastructure for interdisciplinary studies in the genetic epidemiology of colorectal cancer [[3](#_ENREF_3)]. The CCFR includes data from approximately 30,500 total subjects (10,500 probands, and 20,000 unaffected and affected relatives and unrelated controls). Cases and controls, age 20 to 74 years, were recruited at the six participating centers beginning in 1998. CCFR implemented a standardized questionnaire that is administered to all participants, and includes established and suspected risk factors for colorectal cancer, which includes questions on medical history and medication use, reproductive history (for female participants), family history, physical activity, demographics, alcohol and tobacco use, and dietary factors. For genome-wide interaction analysis we only included the CCFR Set 1 scan, which has been described previously [[4](#_ENREF_4)], includes population-based cases and age-matched controls from the three population-based centers: Seattle, Toronto and Australia. Cases were genetically enriched by over-sampling those with a young age at onset or positive family history. Controls were matched to cases on age and sex. All cases and controls were self-reported as White, which was confirmed with genotype data.

**Darmkrebs: Chancen der Verhütung durch Screening (DACHS)** [[5](#_ENREF_5), [6](#_ENREF_6)]. This German study was initiated as a large population-based case-control study in 2003 in the Rhine-Neckar-Odenwald region (southwest region of Germany) to assess the potential of endoscopic screening for reduction of colorectal cancer risk and to investigate etiologic determinants of disease, particularly lifestyle/environmental factors and genetic factors. Cases with a first diagnosis of invasive colorectal cancer (ICO-10 codes C18-C20) who were at least 30 years of age (no upper age limit), German speaking, a resident in the study region, and mentally and physically able to participate in a one-hour interview, were recruited by their treating physicians either in the hospital a few days after surgery, or by mail after discharge from the hospital. Cases were confirmed based on histologic reports and hospital discharge letters following diagnosis of colorectal cancer. All hospitals treating colorectal cancer patients in the study region participated. Based on estimates from population-based cancer registries, more than 50% of all potentially eligible patients with incident colorectal cancer in the study region were included. Community-based controls were randomly selected from population registries, employing frequency matching with respect to age (5-year groups), sex, and county of residence. Controls with a history of colorectal cancer were excluded. Controls were contacted by mail and follow-up calls. The participation rate was 51%. During an in-person interview, data were collected on demographics, medical history, family history of CRC, and various life-style factors, as were blood and mouthwash samples. The Set 1 scan consisted of a subset of participants recruited up to 2007, and controls were frequency matched to cases on age, gender, and county of residence. The Set 2 scan consisted of additional subjects that were recruited up to 2010 as part of this ongoing study.

**Diet, Activity, and Lifestyle Study (DALS**) [[7](#_ENREF_7)]. DALS is a population-based case-control study of colon cancer. Participants were recruited between 1991 and 1994 from three locations: the Kaiser Permanente Medical Care Program (KPMCP) of Northern California, an eight-county area in Utah, and the metropolitan Twin Cities area of Minnesota. Eligibility criteria for cases included age at diagnosis between 30 and 79 years, diagnosis with first primary colon cancer (ICD-O-2 codes 18.0 and 18.2-18.9) between October 1^st^ 1991 and September 30^th^ 1994, English speaking, and competency to complete the interview. Individuals with cancer of the rectosigmoid junction or rectum were excluded, as were those with a pathology report noting familial adenomatous polyposis, Crohn’s disease, or ulcerative colitis. A rapid-reporting system was used to identify all incident cases of colon cancer resulting in the majority of cases being interviewed within four months of diagnosis. Controls from KPMCP were randomly selected from membership lists. In Utah, controls under 65 years of age were randomly selected through random-digit dialing and driver license lists. Controls, 65 years of age and older, were randomly selected from Health Care Financing Administration lists. In Minnesota, controls were identified from Minnesota driver’s license or state ID lists. Cases and controls were matched to cases by 5-year age groups and sex. The Set I scan consisted of a subset of the study designed above, from Utah, Minnesota, and KPMCP, and was restricted to subjects who self-reported as White non-Hispanic. The Set 2 scan consisted of subjects from Utah and Minnesota that were not genotyped in Set 1. Set 2 was restricted to subjects who self-reported as White non-Hispanic and those that had appropriate consent to post data to dbGaP.

**Hawaii Colorectal Cancer Studies 2 & 3 (Colo2&3)** [[8](#_ENREF_8)]. Patients with colorectal cancer were identified through the rapid reporting system of the Hawaii SEER registry and consisted of all Japanese, Caucasian, and Native Hawaiian residents of Oahu who were newly diagnosed with an adenocarcinoma of the colon or rectum between January 1994 and August 1998. Control subjects were selected from participants in an on-going population-based health survey conducted by the Hawaii State Department of Health and from Health Care Financing Administration participants. Controls were matched to cases by sex, ethnicity, and age (within two years). Personal interviews were obtained from 768 matched pairs, resulting in a participation rate of 58.2% for cases and 53.2% for controls. A questionnaire, administered during an in-person interview, included questions about demographics, lifetime history of tobacco, alcohol use, aspirin use, physical activity, personal medical history, family history of colorectal cancer, height and weight, diet (FFQ), and postmenopausal hormone use. A blood sample was obtained from 548 (71%) of interviewed cases and 662 (86%) of interviewed controls. SEER staging information was extracted from the Hawaii Tumor Registry. In GECCO, self-reported Caucasian subjects with DNA, and clinical and epidemiologic data were selected for genotyping.

**Health Professionals Follow-up Study (HPFS)** [[9](#_ENREF_9)]. The HPFS is a parallel prospective study to the Nurses’ Health Study (NHS). The HPFS cohort comprises 51,529 men who, in 1986, responded to a mailed questionnaire. The participants are U.S. male dentists, optometrists, osteopaths, podiatrists, pharmacists, and veterinarians born between 1910 and 1946. Participants have provided information on health related exposures, including: current and past smoking history, age, weight, height, diet, physical activity, aspirin use, and family history of colorectal cancer. Colorectal cancer and other outcomes were reported by participants or next-of-kin and followed up through review of the medical and pathology record by physicians. Overall, more than 97% of self-reported colorectal cancers were confirmed by medical record review. Information was abstracted on histology and primary location. Incident cases are defined as those occurring after the subject provided the blood sample. Prevalent cases are defined as those occurring after enrollment in the study, but prior to the subject providing the blood sample. Follow-up has been excellent, with 94% of the men responding to date. Colorectal cancer cases were ascertained through January 1, 2008. In 1993-95, 18,825 men in HPFS mailed in blood samples by overnight courier which were aliquoted into buffy coat and stored in liquid nitrogen. In 2001-04, 13,956 men in HPFS who had not previously provided a blood sample mailed in a "swish-and-spit" sample of buccal cells. Incident cases are defined as those occurring after the subject provided a blood or buccal sample. Prevalent cases are defined as those occurring after enrollment in the study in 1986, but prior to the subject providing either a blood or buccal sample. After excluding participants with histories of cancer (except non-melanoma skin), ulcerative colitis, or familial polyposis, two case-control sets were constructed from which DNA was isolated from either buffy coat or buccal cells for genotyping: 1) a case-control set with cases of colorectal cancer matched to randomly selected controls who provided a blood sample and were free of colorectal cancer at the same time the colorectal cancer was diagnosed in the cases; 2) a case-control set with cases of colorectal cancer matched to randomly selected controls who provided a buccal sample and were free of colorectal cancer at the same time the colorectal cancer was diagnosed in the case. For both case-control sets, matching criteria included year of birth (within 1 year) and month/year of blood or buccal cell sampling (within six months). Cases were pair matched 1:1, 1:2, or 1:3 with a control participant(s).

In addition to colorectal cancer cases and controls, a set of adenoma cases and matched controls with available DNA from buffy coat were selected for genotyping. Over follow-up, data were collected on endoscopic screening practices and, if individuals have been diagnosed with polyp, the polyps were confirmed to be adenomatous by medical record review. Adenoma cases were ascertained through January 1, 2008. A separate case-control set was constructed of participants diagnosed with advanced adenoma matched to control participants who underwent a lower endoscopy in the same time period and did not have an adenoma. Advanced adenoma was defined as an adenoma >=1 cm in diameter and / or with tubulovillous, villous, or high-grade dysplasia / carcinoma-in-situ histology. Matching criteria included year of birth (within one year) and month/year of blood sampling (within six months), the reason for their lower endoscopy (screening, family history, or symptoms) and the time period of any prior endoscopy (within two years). Controls matched to cases with a distal adenoma either had a negative sigmoidoscopy or colonoscopy exam and controls matched to cases with proximal adenoma all had a negative colonoscopy.

**Multiethnic Cohort Study (MEC)** [[10](#_ENREF_10)]. MEC was initiated in 1993 to investigate the impact of dietary and environmental factors on major chronic diseases, particularly cancer, in ethnically diverse populations in Hawai’i and California. The study recruited 96,810 men and 118,441 women aged 45 to 75 years between 1993 and 1996. Incident colorectal cancer cases occurring since January 1995, and controls were contacted for blood or saliva samples. The median interval between diagnosis and blood draw was 14 months (interquartile range, 10-19) among cases and the participation rate 74%. A sample of cohort participants was randomly selected to serve as controls at the onset of the nested case-control study (participation rate 66%). The selection was stratified by sex, age, and race/ethnicity. Colorectal cancer cases are identified through the Rapid Reporting System of the Hawai’i Tumor Registry and through quarterly linkage to the Los Angeles County Cancer Surveillance Program. Both registries are members of SEER. In GECCO, self-reported White subjects from the nested case-control study described above with DNA, and clinical and epidemiologic data were selected for genotyping

**Nurses’ Health Study (NHS)** [[11](#_ENREF_11)]. The NHS cohort began in 1976 when 121,700 married female registered nurses aged 30 to 55 years returned the initial questionnaire that ascertained a variety of important health-related exposures. Since 1976, follow-up questionnaires have been mailed every two years. Colorectal cancer and other outcomes were reported by participants or next-of-kin and followed up through review of the medical and pathology record by physicians. Overall, more than 97% of self-reported colorectal cancers were confirmed by medical-record review. Information was abstracted on histology and primary location. Follow-up has been high: as a proportion of the total possible follow-up time, follow-up has been over 92%. Colorectal cancer cases were ascertained through June 1, 2008. In 1989-90, 32,826 women in NHS I, mailed in blood samples by overnight courier which were aliquoted into buffy coat and stored in liquid nitrogen. In 2001-04, 29,684 women in NHS I who did not previously provide a blood sample mailed in a "swish-and-spit" sample of buccal cells. Incident cases are defined as those occurring after the subject provided a blood or buccal sample. Prevalent cases are defined as those occurring after enrollment in the study in 1976, but prior to the subject providing either a blood or buccal sample. After excluding participants with histories of cancer (except non-melanoma skin), ulcerative colitis, or familial polyposis, we constructed two case-control sets from which DNA was isolated from either buffy coat or buccal cells for genotyping: 1) a case-control set with cases of colorectal cancer matched to randomly selected controls who provided a blood sample and were free of colorectal cancer at the same time the colorectal cancer was diagnosed in the case; 2) a case-control set with cases of colorectal cancer matched to randomly selected controls who provided a buccal sample and were free of colorectal cancer at the same time the colorectal cancer was diagnosed in the cases. For both case-control sets, matching criteria included year of birth (within one year) and month / year of blood or buccal cell sampling (within six months). Cases were pair matched 1:1, 1:2, or 1:3 with a control participant(s).

In addition to colorectal cancer cases and controls, a set of adenoma cases and matched controls with available DNA from buffy coat were selected for genotyping. Over follow-up, data were collected on endoscopic screening practices and, if individuals have been diagnosed with polyp, the polyps confirmed to be adenomatous by medical record review. Adenoma cases were ascertained through June 1, 2008. A separate case-control set was constructed of participants diagnosed with advanced adenoma matched to control participants who underwent a lower endoscopy in the same time period and did not have an adenoma. Advanced adenoma was defined as an adenoma > 1 cm in diameter and / or with tubulovillous, villous, or high-grade dysplasia / carcinoma-in-situ histology. Matching criteria included year of birth (within one year) and month/year of blood sampling (within six months), the reason for their lower endoscopy (screening, family history, or symptoms) and the time period of any prior endoscopy (within two years). Controls matched to cases with a distal adenoma either had a negative sigmoidoscopy or colonoscopy exam and controls matched to cases with proximal adenoma all had a negative colonoscopy.

**Ontario Familial Colorectal Cancer Registry** (**OFCCR)**. A subset of the Assessment of Risk in Colorectal Tumours in Canada (ARCTIC) from the Ontario Registry for Studies of Familial Colorectal Cancer (OFCCR) was used. Both the case-control study [[12](#_ENREF_12)] and the OFCCR [[13](#_ENREF_13)] have been described in detail previously, as have GWAS results [[14](#_ENREF_14)]. In brief, cases were confirmed incident colorectal cancer (CRC) cases ages 20 to 74 years, residents of Ontario identified through comprehensive registry and diagnosed between July 1997 and June 2000. Population-based controls were randomly selected among Ontario residents (random-digit-dialing and listing of all Ontario residents), and matched by sex and 5-year age groups. A total of 1,236 CRC cases and 1,223 controls were successfully genotyped on at least one of the Illumina 1536 GoldenGate assay, the Affymetrix GeneChip® Human Mapping 100K and 500K Array Set, and a 10K non-synonymous SNP chip. Analysis was based on a set of unrelated subjects who were non-Hispanic, White by self-report or by investigation of genetic ancestry. We further excluded subjects if there was a sample mix-up, if they were missing epidemiologic questionnaire data, if they were appendix cases, or if they were overlapped with the Colon Cancer Family Registry GWAS (see above). Additionally, only samples genotyped on the Affymetrix GeneChip® 500K Array were utilized in order to avoid coverage issues in imputation.

**Physician’s Health Study (PHS**) [[15](#_ENREF_15), [16](#_ENREF_16)]. The PHS was established as a randomized, double-blind, placebo-controlled trial of aspirin and ß-carotene among 22,071 healthy U.S. male physicians, between 40 and 84 years of age in 1982. Participants completed two mailed questionnaires before being randomly assigned, additional questionnaires at six and 12 months, and questionnaires annually thereafter. In addition, participants were sent postcards at six months to ascertain status. From August 1982 to December 1984, 14,916 baseline blood samples were collected from the physicians during the run-in phase before randomization. When participants report a diagnosis of cancer, medical records and pathology reports are reviewed by study physicians who are blinded to exposure data. Among those who provided baseline blood samples, colorectal cases were ascertained through March 31, 2008, and controls were matched on age (within one year for younger participants, up to five years for older participants) and smoking status (never, past, current). Cases were “pair” matched 1:1, 1:2 or 1:3 with a control participant(s). Due to DNA availability samples were genotyped in two batches on the same platform at the same genotyping center at different time points.

**Prostate, Lung, Colorectal, and Ovarian Cancer Screening Trial (PLCO)**. PLCO enrolled 154,934 participants (men and women, aged between 55 and 74 years) at ten centers into a large, randomized, two-arm trial to determine the effectiveness of screening to reduce cancer mortality. Sequential blood samples were collected from participants assigned to the screening arm. Participation was 93% at the baseline blood draw. In the observational (control) arm, buccal cells were collected via mail using the “swish-and-spit” protocol and participation rate was 65%. Details of this study have been previously described [[17](#_ENREF_17), [18](#_ENREF_18)] and are available online (http://dcp.cancer.gov/plco).

The Set 1 GWAS included a subset of 577 colon cancer cases self-reported as being non-Hispanic White with available DNA samples, questionnaire data, and appropriate consent for ancillary epidemiologic studies. Cases were excluded if they had a history of inflammatory bowel disease, polyps, polyposis syndrome or cancer (excluding basal or squamous cell skin cancer). Sex- and age matched controls were selected among participants from the Cancer Genetic Markers of Susceptibility (CGEMS) prostate cancer scan [[19](#_ENREF_19), [20](#_ENREF_20)] and the GWAS of Lung Cancer and Smoking [[21](#_ENREF_21)] and the Pancreatic Cancer Cohort Consortium (PanScan) [[22](#_ENREF_22), [23](#_ENREF_23)] along with an additional 92 non-Hispanic White female controls that were genotyped together with the cases. For the Set 2 scan, cases were colorectal cancers from both arms of the trial, which were not already included in Set 1. Samples were excluded if participants did not sign appropriate consents, if DNA was unavailable, if baseline questionnaire data with follow-up were unavailable, if they had a history of colon cancer prior to the trial, if they were a rare cancer, and if they were already in colon GWAS, or if they were a control in the prostate or lung populations. Controls were frequency matched 1:1 to cases without replacement, and cases were not eligible to be controls. Matching criteria were age at enrollment (two year blocks), enrollment date (two year blocks), sex, race / ethnicity, trial arm, and study year of diagnosis (i.e. controls must be cancer free into the case's year of diagnosis).

**Postmenopausal Hormones Supplementary Study to the Colon Cancer Family Registry (PMH-CCFR)** [[24](#_ENREF_24)]. Eligible case patients included all female residents, ages 50 to 74 years, residing in the 13 counties in Washington State reporting to the Cancer Surveillance SEER program, who were newly diagnosed with invasive colorectal adenocarcinoma (ICD-O C18.0, C18.2-.9, C19.9, C20.0-.9) between October 1998 and February 2002. Eligibility for all individuals was limited to those who were English-speaking with available telephone numbers, in which they could be contacted. On average, cases were identified within four months of diagnosis. The overall response proportion of eligible cases identified was 73%. Community-based controls were randomly selected according to age distribution (in 5-year age intervals) of the eligible cases by using lists of licensed drivers from the Washington State Department of Licensing for individuals, ages 50 to 64 years, and rosters from the Health Care Financing Administration (now the Centers for Medicare and Medicaid) for individuals older than 64 years. The overall response proportion of eligible controls was 66%. In GECCO, samples with sufficient DNA extracted from blood were genotyped. Only participants that were not part of the CCFR Seattle site were included in the sample set.

**VITamins And Lifestyle (VITAL).** The VITamins And Lifestyle (VITAL) cohort comprises 77,721 Washington State men and women aged 50 to 76 years, recruited from 2000 to 2002 to investigate the association of supplement use and lifestyle factors with cancer risk. Subjects were recruited by mail, from October 2000 to December 2002, using names purchased from a commercial mailing list. All subjects competed a 24 page questionnaire and buccal-cell specimens for DNA was self-collected by 70% of the participants. Subjects are followed for cancer by linkage to the western Washington SEER cancer registry and are censored when they move out of the area covered by the registry or at time of death. Details of this study have been previously described [[25](#_ENREF_25)]. In GECCO, a nested case-control set was genotyped. Samples included, colorectal cancer cases with DNA, excluding subject with colorectal cancer before baseline, in situ cases, (large cell) neuroendocrine carcinoma, squamous cell carcinoma, carcinoid tumor, Goblet cell carcinoid, any type of lymphoma, including non-Hodgkin, Mantle cell, large B-cell, or follicular lymphoma. Controls were matched on age at enrollment (within one year), enrollment date (within one year), sex, and race / ethnicity. One control was randomly selected per case among all controls that matched on the four factors above and where the control follow-up time was greater than follow-up time of the case until diagnosis.

**Women’s Health Initiative (WHI).** WHI is a long-term health study of 161,808 post-menopausal women aged 50 to 79 years at 40 clinical centers throughout the U.S. WHI comprises a Clinical Trial (CT) arm, an Observational Study (OS) arm, and several extension studies. The details of WHI have been previously described [[26](#_ENREF_26), [27](#_ENREF_27)] and are available online (https://cleo.whi.org/SitePages/Home.aspx). In GECCO, Set 1 cases were selected from the September 12, 2005 database and comprised centrally adjudicated colon cancer cases from the Observational Study (OS) who self-reported as White. Controls were first selected among controls previously genotyped as part of a Hip Fracture GWAS conducted within the WHI OS and matched to cases on age (within three years) enrollment date (within 365 days), hysterectomy status, and prevalent conditions at baseline. For 37 cases, there was not a control match in the Hip Fracture GWAS. For these participants, we identified a matched control in the WHI OS based on same criteria. In the Set 2 scan, cases were selected from the August 2009 database and were comprised of centrally adjudicated colon and colorectal cancer cases from the OS and CT who were not genotyped in Set 1. In addition, case and control participants were subject to the following exclusion criteria: a prior history of colorectal cancer at baseline, IRB approval not available for data submission into dbGaP, and not sufficient DNA available. Matching criteria included age (within years), race/ethnicity, WHI date (within three years), WHI Calcium and Vitamin D study date (within three years), and randomization arms (OS flag, hormone therapy assignments, dietary modification assignments, calcium/vitamin D assignments). In addition, they were matched on the four regions of randomization centers. Each case was matched with one control (1:1) that exactly met the matching criteria. Control selection was done in a time-forward manner, selecting one control for each case first from the risk set at the time of the case’s event. The matching algorithm was allowed to select the closest match based on a criterion to minimize an overall distance measure [[28](#_ENREF_28)]. Each matching factor was given the same weight. Additional available controls genotyped as part of the Hip Fracture GWAS were included to improve power.

**Genotyping and quality control**

Detailed information on genotyping and quality-control procedures have been described previously [[1](#_ENREF_1), [29](#_ENREF_29)]. All analyses were based on genotype data generated from genome-wide association scans and imputation to 1000 Genomes Project as described in the main text.

CCFR genotyping was based on Illumina Human1M 4. Phase one genotyping of PLCO, WHI, and DALS (PLCO Set 1, WHI Set 1, and DALS Set 1) was done using Illumina HumanHap 550K, 610K, or combined Illumina 300K and 240K, and has been described previously 5. OFCCR was genotyped using Affymetrix platforms 6. ASTERISK, Colo2&3, DACHS Set 1, DALS Set 2, MEC, PMH, PLCO Set 2, VITAL, and WHI Set 2 were genotyped using Illumina HumanCytoSNP. HPFS, NHS, PHS, and DACHS Set 2 were genotyped using Illumina HumanOmniExpress.

DNA was extracted from blood samples or, for a subset of DACHS, HPFS, MEC, NHS, and PLCO samples, and for all VITAL samples, from buccal cells, using conventional methods. All studies included 1 to 6% blinded duplicates to monitor quality of the genotyping. All individual-level genotype data were managed, and underwent quality assurance and quality control (QA/QC) at University of Southern California (CCFR), the Ontario Institute for Cancer Research (OFCCR), the University of Washington Genetics Coordinating Center (HPFS, NHS, PHS, and DACHS Set 2), or the GECCO Coordinating Center at the Fred Hutchinson Cancer Research Center (all other studies). Samples were excluded based on call rate, heterozygosity, unexpected duplicates, gender discrepancy, and unexpectedly high identity-by-descent or unexpected genotype concordance (> 65%) with another individual. Single nucleotide polymorphisms (SNPs) were excluded if they were triallelic, not assigned an rs number, or were reported or observed as not performing consistently across platforms. Additionally, genotyped SNPs were excluded based on call rate (< 98%) and lack of Hardy-Weinberg Equilibrium in controls (HWE, p < 1 x 10-4).

After genotyping, imputation (as detailed in main text), and quality control analyses, a total of 9,108,348 SNPs and 1,276,562 insertions or deletions remained. To evaluate overall performance, we calculated the genomic inflation factor (λ) to measure the over-dispersion of the test-statistics from the genome-wide marginal association tests by dividing the median of the squared Z statistics by 0.455, the median of a chi-squared distribution with 1 degree of freedom. The inflation factor λ was between 0.999 and 1.044 for individual studies based on all SNPs including both directly genotyped and imputed SNPs, indicating little evidence of residual population substructure, cryptic relatedness, or differential genotyping between cases and controls. This result was consistent with the visual inspection of the study-specific Q-Q plots.

**Functional annotation using bioinformatics**

The UCSC Genome Browser [[30](#_ENREF_30)] provides several bioinformatics tools for the functional characterization of candidate regulatory SNPs at disease associated loci. In this analysis several datasets from the ENCODE [[31](#_ENREF_31)] and Roadmap Epigenomic projects [[32](#_ENREF_32)] were queried through the UCSC Genome Browser to predict the regulatory landscape in regions containing any of the following SNPs: 1) the variant showing the strongest association signal (smallest *P*-value) in each region (i.e., top SNP), 2) the GWAS-identified variant in each region (i.e., index SNP), 3) among the top 10 variants with the smallest *P*-values in each region, variants that were correlated (*r^2^*>0.5 in 1000 Genomes European populations) with the index SNP, and 4) any SNP completely correlated (*r^2^*=1 in 1000 Genomes European populations) with any SNP listed in parts 1-3. In addition, we annotated SNP(s) showing the smallest *P*-values after performing conditional analyses that simultaneously included the index SNP(s) in multivariable models. After aligning variants from this SNP list to the reference genome in the UCSC Genome Browser, each variant was annotated with data from DNaseI hypersensitivity assays, ChiPseq assays, and PhastCons 46-way vertebrate evolutionary conservation dataset. We also conducted a binding motif analysis using the HaploReg [[33](#_ENREF_33)] compiled position weight matrix library to identify predicted differences in binding affinity between sequences harboring either the reference or alternate allele. The accumulation of evidence across these datasets was used to assign the relative strength of the functional evidence for each variant (weak, moderate, or strong) as detailed in the main text.

Annotation of variants in genomic regions that do not encode proteins (non -coding regions) assumes that risk variants affect disease by altering levels of transcription through multiple regulatory mechanisms. Using a custom track in the genome browser, a list of correlated variants was aligned with several tracks to provide evidence of enhancer, promoter, insulator and/or silencer activity. Since distal enhancers often facilitate cell-type specific expression, it is helpful to look for evidence in a variety of cell lines in addition to those specific to the trait of interest [[34](#_ENREF_34)]. Therefore, we queried both colorectal cancer cell lines such as CACO2 and HCT116, and a wider variety of cancerous and non-cancerous cell lines to identify SNPs falling in actively regulated regions. It is also of great interest to identify regions that show differential regulatory evidence between normal colon tissue (available through Roadmap) and colon cancer cell lines (available through ENCODE).

The methylation and acetylation of histone proteins changes chromatin accessibility for transcriptional machinery and such marks can serve as a powerful tool for identifying specific regulatory activity [[35](#_ENREF_35)]. ENCODE and Roadmap have assayed many chemical modifications and cell lines to identify promoter, enhancer and repressive marks. For example, the H3K4me1 histone mark is associated with enhancers downstream of transcription start sites and the H3k27Ac histone mark is similarly thought to enhance transcription by blocking the repressive properties of H3K27Me3. Such evidence can help develop specific regulatory hypotheses.

Regulatory regions associate with open chromatin structure, which is accessible to transcriptional machinery and are therefore also susceptible to DNaseI cutting [[36](#_ENREF_36)]. ENCODE has assayed this enzyme in a large collection of cell types and has reported regions of the genome that display DNaseI hypersensitivity. The DNaseI cluster track provides a more precise demarcation of non-specific regulatory regions. However, DNaseI hypersensitivity delivers less insight into the particular activity associated with the region than ChIPseq assays. Therefore, DNaseI hypersensitivity can be used to either identify regions with nonspecific regulation or more precisely define particular regions of interest within a broad ChIPseq signal.

The ChIP-Seq Transcription Factor Binding Site track provides evidence for the binding of specific proteins associated with gene expression. Using the ChIPseq method, this track helps identify the alteration of sites that experimentally bind proteins. For example, CTCF is a transcription factor that assumes multiple forms and can act as an activator, a repressor/silencer, or an insulator [[37](#_ENREF_37)]. When binding chromatic insulators it can prevent interactions between promoters and nearby enhancers or silencers. However, it also mediates long-range chromatin looping, which can bring distal enhancers in proximity of a gene’s promoter.

There are also several other bioinformatics tools that examine the genomic sequence, rather than data from functional assays, to identify regions of functional interest. Although less specific than the ChiP-Seq Transcription Factor Binding Site dataset, the HaploReg database can be used to query a larger collection of position weight matrix libraries, including JASPAR [[38](#_ENREF_38)] and TRANSFAC [[39](#_ENREF_39)], to predict whether a sequence harboring either the reference or alternate allele would exhibit altered affinities in conserved binding motifs for regulatory proteins. In addition, identification of evolutionarily conserved segments has been established as a useful tool to discover functionally important regions [[40](#_ENREF_40)], and we examined these using the PhastCons 46-way vertebrate evolutionary conservation dataset [[41](#_ENREF_41), [42](#_ENREF_42)]. However, data on histone modification and DNaseI hypersensitivity are more robust tools for annotation of non-coding variants because regulatory elements are not always necessary across vertebrate evolution.

Prioritizing variants for further laboratory evaluation **c**an be accomplished by examining the evidence accumulated across these datasets (see details in main text). Combining the strengths and weaknesses of each of these sets can provide *in silico* evidence for regulatory function, and enables selection of strong candidates for more cost- and labor-intensive follow-up studies using reporter gene methods. The Table below summarizes the various datasets and bioinformatics tools used in the present analysis.

Tools for functional annotation of non-coding variants

| Biological dataset/*in silico* tool | Assay/method | Resolution | Genomic class | Description | Functional evidence |
| --- | --- | --- | --- | --- | --- |
| ENCODE/Roadmap H3K4Me1 | ChIP-seq | Broad region | Non-promoter regulatory elements | Identifies DNA regions interacting with the mono-methylation of lysine 4 of the H3 histone. | Associated with downstream enhancers. |
| ENCODE/ Roadmap H3K4Me3 | ChIP-seq | Broad region | Promoter regulatory element | Identifies DNA regions interacting with the tri-methylation of lysine 4 of the H3 histone. | Associated with promoters that are active or accessible for activation. |
| ENCODE/ Roadmap H3K27Ac | ChIP-seq | Broad region | Non-promoter regulatory elements | Identifies DNA regions interacting with the acetylation of lysine 27 of the H3 histone. | Enhances transcription possibly by blocking the spread of the repressive histone mark H3K27Me3. |
| ENCODE DNase Clusters | Digital DNaseI hypersensitivity clusters | Precise region | Nonspecific regulatory element | Maps nonspecific regulatory regions in a large collection of cell lines. | Open chromatin structure corresponds to both enhancers and promoters |
| ENCODE Transcription Factor Binding Site | ChIP-seq | Precise region | Protein bound DNA | Identifies regions bound by specific transcription factors. | Activators can recruit RNA polymerase, repressors suppress transcription, and insulators block the activity of nearby activators or repressors. |
| ENCODE CTCF Binding | ChIP-seq | Precise region | Insulated element or loop DNA | Identifies regions bound by CTCF, which functions as both a transcriptional activator and an insulator. | Mediates long-range chromatin looping, which can bring enhancers or insulators to promoters. |
| Haploreg ‘Motifs Changed’ | Position weight matrix from JASPAR, TRANSFAC, and literature | Nucleotide | Altered binding motif | Change in log-odds score was calculated for a motif overlapping a SNP in position weight matrices that passed a threshold of p<4E-07 | Identification of binding motifs that are altered by SNPs. |
| Vertebrate Conservation | PhastCons | Nucleotide | Conserved element | Multiple alignments of 46 vertebrate species. Estimates the probability that each nucleotide belongs to a conserved element. | Identification of evolutionarily conserved segments of homology, potentially identifying a functionally important region. |

**REFERENCES**

1. Peters U, Jiao S, Schumacher FR, Hutter CM, Aragaki AK, Baron JA, et al. Identification of Genetic Susceptibility Loci for Colorectal Tumors in a Genome-wide Meta-analysis. Gastroenterology. 2012. Epub 2012/12/26. doi: 10.1053/j.gastro.2012.12.020. PubMed PMID: 23266556.

2. Kury S, Buecher B, Robiou-du-Pont S, Scoul C, Sebille V, Colman H, et al. Combinations of cytochrome P450 gene polymorphisms enhancing the risk for sporadic colorectal cancer related to red meat consumption. Cancer epidemiology, biomarkers & prevention : a publication of the American Association for Cancer Research, cosponsored by the American Society of Preventive Oncology. 2007;16(7):1460-7. Epub 2007/07/14. doi: 10.1158/1055-9965.EPI-07-0236. PubMed PMID: 17627011.

3. Newcomb PA, Baron J, Cotterchio M, Gallinger S, Grove J, Haile R, et al. Colon Cancer Family Registry: an international resource for studies of the genetic epidemiology of colon cancer. Cancer epidemiology, biomarkers & prevention : a publication of the American Association for Cancer Research, cosponsored by the American Society of Preventive Oncology. 2007;16(11):2331-43. Epub 2007/11/06. doi: 10.1158/1055-9965.EPI-07-0648. PubMed PMID: 17982118.

4. Figueiredo JC, Lewinger JP, Song C, Campbell PT, Conti DV, Edlund CK, et al. Genotype-environment interactions in microsatellite stable/microsatellite instability-low colorectal cancer: results from a genome-wide association study. Cancer epidemiology, biomarkers & prevention : a publication of the American Association for Cancer Research, cosponsored by the American Society of Preventive Oncology. 2011;20(5):758-66. Epub 2011/03/02. doi: 10.1158/1055-9965.EPI-10-0675. PubMed PMID: 21357381; PubMed Central PMCID: PMC3089660.

5. Brenner H, Chang-Claude J, Seiler CM, Rickert A, Hoffmeister M. Protection from colorectal cancer after colonoscopy: a population-based, case-control study. Annals of internal medicine. 2011;154(1):22-30. Epub 2011/01/05. doi: 10.1059/0003-4819-154-1-201101040-00004. PubMed PMID: 21200035.

6. Lilla C, Verla-Tebit E, Risch A, Jager B, Hoffmeister M, Brenner H, et al. Effect of NAT1 and NAT2 genetic polymorphisms on colorectal cancer risk associated with exposure to tobacco smoke and meat consumption. Cancer epidemiology, biomarkers & prevention : a publication of the American Association for Cancer Research, cosponsored by the American Society of Preventive Oncology. 2006;15(1):99-107. Epub 2006/01/26. doi: 10.1158/1055-9965.EPI-05-0618. PubMed PMID: 16434594.

7. Slattery ML, Potter J, Caan B, Edwards S, Coates A, Ma KN, et al. Energy balance and colon cancer--beyond physical activity. Cancer research. 1997;57(1):75-80. Epub 1997/01/01. PubMed PMID: 8988044.

8. Le Marchand L, Hankin JH, Wilkens LR, Pierce LM, Franke A, Kolonel LN, et al. Combined effects of well-done red meat, smoking, and rapid N-acetyltransferase 2 and CYP1A2 phenotypes in increasing colorectal cancer risk. Cancer epidemiology, biomarkers & prevention : a publication of the American Association for Cancer Research, cosponsored by the American Society of Preventive Oncology. 2001;10(12):1259-66. Epub 2001/12/26. PubMed PMID: 11751443.

9. Rimm EB, Stampfer MJ, Colditz GA, Chute CG, Litin LB, Willett WC. Validity of self-reported waist and hip circumferences in men and women. Epidemiology. 1990;1(6):466-73. Epub 1990/11/01. PubMed PMID: 2090285.

10. Kolonel LN, Henderson BE, Hankin JH, Nomura AM, Wilkens LR, Pike MC, et al. A multiethnic cohort in Hawaii and Los Angeles: baseline characteristics. American journal of epidemiology. 2000;151(4):346-57. Epub 2000/03/01. PubMed PMID: 10695593.

11. Belanger CF, Hennekens CH, Rosner B, Speizer FE. The nurses' health study. The American journal of nursing. 1978;78(6):1039-40. Epub 1978/06/01. PubMed PMID: 248266.

12. Cotterchio M, Manno M, Klar N, McLaughlin J, Gallinger S. Colorectal screening is associated with reduced colorectal cancer risk: a case-control study within the population-based Ontario Familial Colorectal Cancer Registry. Cancer causes & control : CCC. 2005;16(7):865-75. Epub 2005/09/01. doi: 10.1007/s10552-005-2370-3. PubMed PMID: 16132797.

13. Cotterchio M, McKeown-Eyssen G, Sutherland H, Buchan G, Aronson M, Easson AM, et al. Ontario familial colon cancer registry: methods and first-year response rates. Chronic diseases in Canada. 2000;21(2):81-6. Epub 2000/09/29. PubMed PMID: 11007659.

14. Zanke BW, Greenwood CM, Rangrej J, Kustra R, Tenesa A, Farrington SM, et al. Genome-wide association scan identifies a colorectal cancer susceptibility locus on chromosome 8q24. Nature genetics. 2007;39(8):989-94. Epub 2007/07/10. doi: 10.1038/ng2089. PubMed PMID: 17618283.

15. Hennekens CH, Eberlein K. A randomized trial of aspirin and beta-carotene among U.S. physicians. Preventive medicine. 1985;14(2):165-8. Epub 1985/03/01. PubMed PMID: 3900975.

16. Christen WG, Gaziano JM, Hennekens CH. Design of Physicians' Health Study II--a randomized trial of beta-carotene, vitamins E and C, and multivitamins, in prevention of cancer, cardiovascular disease, and eye disease, and review of results of completed trials. Annals of epidemiology. 2000;10(2):125-34. Epub 2000/02/26. PubMed PMID: 10691066.

17. Prorok PC, Andriole GL, Bresalier RS, Buys SS, Chia D, Crawford ED, et al. Design of the Prostate, Lung, Colorectal and Ovarian (PLCO) Cancer Screening Trial. Controlled clinical trials. 2000;21(6 Suppl):273S-309S. Epub 2001/02/24. PubMed PMID: 11189684.

18. Gohagan JK, Prorok PC, Hayes RB, Kramer BS, Prostate LC, Ovarian Cancer Screening Trial Project T. The Prostate, Lung, Colorectal and Ovarian (PLCO) Cancer Screening Trial of the National Cancer Institute: history, organization, and status. Controlled clinical trials. 2000;21(6 Suppl):251S-72S. Epub 2001/02/24. PubMed PMID: 11189683.

19. Ci X, Li B, Ma X, Kong F, Zheng C, Bjorkholm M, et al. Bortezomib-mediated down-regulation of telomerase and disruption of telomere homeostasis contributes to apoptosis of malignant cells. Oncotarget. 2015;6(35):38079-92. doi: 10.18632/oncotarget.5752. PubMed PMID: 26472030; PubMed Central PMCID: PMC4741985.

20. Yeager M, Chatterjee N, Ciampa J, Jacobs KB, Gonzalez-Bosquet J, Hayes RB, et al. Identification of a new prostate cancer susceptibility locus on chromosome 8q24. Nature genetics. 2009;41(10):1055-7. Epub 2009/09/22. doi: 10.1038/ng.444. PubMed PMID: 19767755; PubMed Central PMCID: PMC3430510.

21. Landi MT, Chatterjee N, Yu K, Goldin LR, Goldstein AM, Rotunno M, et al. A genome-wide association study of lung cancer identifies a region of chromosome 5p15 associated with risk for adenocarcinoma. American journal of human genetics. 2009;85(5):679-91. Epub 2009/10/20. doi: 10.1016/j.ajhg.2009.09.012. PubMed PMID: 19836008; PubMed Central PMCID: PMC2775843.

22. Amundadottir L, Kraft P, Stolzenberg-Solomon RZ, Fuchs CS, Petersen GM, Arslan AA, et al. Genome-wide association study identifies variants in the ABO locus associated with susceptibility to pancreatic cancer. Nature genetics. 2009;41(9):986-90. Epub 2009/08/04. doi: 10.1038/ng.429. PubMed PMID: 19648918; PubMed Central PMCID: PMC2839871.

23. Petersen GM, Amundadottir L, Fuchs CS, Kraft P, Stolzenberg-Solomon RZ, Jacobs KB, et al. A genome-wide association study identifies pancreatic cancer susceptibility loci on chromosomes 13q22.1, 1q32.1 and 5p15.33. Nature genetics. 2010;42(3):224-8. Epub 2010/01/27. doi: 10.1038/ng.522. PubMed PMID: 20101243; PubMed Central PMCID: PMC2853179.

24. Newcomb PA, Zheng Y, Chia VM, Morimoto LM, Doria-Rose VP, Templeton A, et al. Estrogen plus progestin use, microsatellite instability, and the risk of colorectal cancer in women. Cancer research. 2007;67(15):7534-9. Epub 2007/08/03. doi: 10.1158/0008-5472.CAN-06-4275. PubMed PMID: 17671225.

25. White E, Patterson RE, Kristal AR, Thornquist M, King I, Shattuck AL, et al. VITamins And Lifestyle cohort study: study design and characteristics of supplement users. American journal of epidemiology. 2004;159(1):83-93. Epub 2003/12/25. PubMed PMID: 14693663.

26. Hays J, Hunt JR, Hubbell FA, Anderson GL, Limacher M, Allen C, et al. The Women's Health Initiative recruitment methods and results. Annals of epidemiology. 2003;13(9 Suppl):S18-77. Epub 2003/10/25. PubMed PMID: 14575939.

27. Design of the Women's Health Initiative clinical trial and observational study. The Women's Health Initiative Study Group. Controlled clinical trials. 1998;19(1):61-109. Epub 1998/03/11. PubMed PMID: 9492970.

28. Bergstralh EJ, Kosanke JL. Computerized matching of cases to controls. 56 ed. Department of Health Sciences Research, Mayo Clinic, Rochester MN: 1995.

29. Hutter CM, Chang-Claude J, Slattery ML, Pflugeisen BM, Lin Y, Duggan D, et al. Characterization of gene-environment interactions for colorectal cancer susceptibility loci. Cancer research. 2012;72(8):2036-44. Epub 2012/03/01. doi: 10.1158/0008-5472.CAN-11-4067. PubMed PMID: 22367214; PubMed Central PMCID: PMC3374720.

30. Kent WJ, Sugnet CW, Furey TS, Roskin KM, Pringle TH, Zahler AM, et al. The human genome browser at UCSC. Genome research. 2002;12(6):996-1006. Epub 2002/06/05. doi: 10.1101/gr.229102. Article published online before print in May 2002. PubMed PMID: 12045153; PubMed Central PMCID: PMC186604.

31. Consortium EP, Birney E, Stamatoyannopoulos JA, Dutta A, Guigo R, Gingeras TR, et al. Identification and analysis of functional elements in 1% of the human genome by the ENCODE pilot project. Nature. 2007;447(7146):799-816. Epub 2007/06/16. doi: 10.1038/nature05874. PubMed PMID: 17571346; PubMed Central PMCID: PMC2212820.

32. Bernstein BE, Stamatoyannopoulos JA, Costello JF, Ren B, Milosavljevic A, Meissner A, et al. The NIH Roadmap Epigenomics Mapping Consortium. Nature biotechnology. 2010;28(10):1045-8. Epub 2010/10/15. doi: 10.1038/nbt1010-1045. PubMed PMID: 20944595; PubMed Central PMCID: PMC3607281.

33. Ward LD, Kellis M. HaploReg: a resource for exploring chromatin states, conservation, and regulatory motif alterations within sets of genetically linked variants. Nucleic acids research. 2012;40(Database issue):D930-4. Epub 2011/11/09. doi: 10.1093/nar/gkr917. PubMed PMID: 22064851; PubMed Central PMCID: PMC3245002.

34. Chepelev I, Wei G, Wangsa D, Tang Q, Zhao K. Characterization of genome-wide enhancer-promoter interactions reveals co-expression of interacting genes and modes of higher order chromatin organization. Cell research. 2012;22(3):490-503. Epub 2012/01/25. doi: 10.1038/cr.2012.15. PubMed PMID: 22270183; PubMed Central PMCID: PMC3292289.

35. Smallwood A, Ren B. Genome organization and long-range regulation of gene expression by enhancers. Current opinion in cell biology. 2013;25(3):387-94. Epub 2013/03/08. doi: 10.1016/j.ceb.2013.02.005. PubMed PMID: 23465541.

36. Thurman RE, Rynes E, Humbert R, Vierstra J, Maurano MT, Haugen E, et al. The accessible chromatin landscape of the human genome. Nature. 2012;489(7414):75-82. Epub 2012/09/08. doi: 10.1038/nature11232. PubMed PMID: 22955617; PubMed Central PMCID: PMC3721348.

37. Sanyal A, Lajoie BR, Jain G, Dekker J. The long-range interaction landscape of gene promoters. Nature. 2012;489(7414):109-13. Epub 2012/09/08. doi: 10.1038/nature11279. PubMed PMID: 22955621; PubMed Central PMCID: PMC3555147.

38. Portales-Casamar E, Thongjuea S, Kwon AT, Arenillas D, Zhao X, Valen E, et al. JASPAR 2010: the greatly expanded open-access database of transcription factor binding profiles. Nucleic acids research. 2010;38(Database issue):D105-10. Epub 2009/11/13. doi: 10.1093/nar/gkp950. PubMed PMID: 19906716; PubMed Central PMCID: PMC2808906.

39. Fu Y, Weng Z. Improvement of TRANSFAC matrices using multiple local alignment of transcription factor binding site sequences. Conference proceedings : Annual International Conference of the IEEE Engineering in Medicine and Biology Society IEEE Engineering in Medicine and Biology Society Conference. 2004;4:2856-9. Epub 2007/02/03. doi: 10.1109/IEMBS.2004.1403814. PubMed PMID: 17270873.

40. Lehmann KV, Chen T. Exploring functional variant discovery in non-coding regions with SInBaD. Nucleic acids research. 2013;41(1):e7. Epub 2012/09/04. doi: 10.1093/nar/gks800. PubMed PMID: 22941663; PubMed Central PMCID: PMC3592431.

41. Pollard KS, Hubisz MJ, Rosenbloom KR, Siepel A. Detection of nonneutral substitution rates on mammalian phylogenies. Genome research. 2010;20(1):110-21. Epub 2009/10/28. doi: 10.1101/gr.097857.109. PubMed PMID: 19858363; PubMed Central PMCID: PMC2798823.

42. Siepel A, Bejerano G, Pedersen JS, Hinrichs AS, Hou M, Rosenbloom K, et al. Evolutionarily conserved elements in vertebrate, insect, worm, and yeast genomes. Genome research. 2005;15(8):1034-50. Epub 2005/07/19. doi: 10.1101/gr.3715005. PubMed PMID: 16024819; PubMed Central PMCID: PMC1182216.

43. Houlston RS, Cheadle J, Dobbins SE, Tenesa A, Jones AM, Howarth K, et al. Meta-analysis of three genome-wide association studies identifies susceptibility loci for colorectal cancer at 1q41, 3q26.2, 12q13.13 and 20q13.33. Nature genetics. 2010;42(11):973-7. Epub 2010/10/26. doi: 10.1038/ng.670. PubMed PMID: 20972440.

44. Jia WH, Zhang B, Matsuo K, Shin A, Xiang YB, Jee SH, et al. Genome-wide association analyses in east Asians identify new susceptibility loci for colorectal cancer. Nature genetics. 2012. Epub 2012/12/25. doi: 10.1038/ng.2505. PubMed PMID: 23263487.

45. Dunlop MG, Dobbins SE, Farrington SM, Jones AM, Palles C, Whiffin N, et al. Common variation near CDKN1A, POLD3 and SHROOM2 influences colorectal cancer risk. Nature genetics. 2012;44(7):770-6. Epub 2012/05/29. doi: 10.1038/ng.2293. PubMed PMID: 22634755.

46. Tomlinson IP, Webb E, Carvajal-Carmona L, Broderick P, Howarth K, Pittman AM, et al. A genome-wide association study identifies colorectal cancer susceptibility loci on chromosomes 10p14 and 8q23.3. Nature genetics. 2008;40(5):623-30. Epub 2008/04/01. doi: 10.1038/ng.111. PubMed PMID: 18372905.

47. Tomlinson I, Webb E, Carvajal-Carmona L, Broderick P, Kemp Z, Spain S, et al. A genome-wide association scan of tag SNPs identifies a susceptibility variant for colorectal cancer at 8q24.21. Nature genetics. 2007;39(8):984-8. Epub 2007/07/10. doi: 10.1038/ng2085. PubMed PMID: 17618284.

48. Haiman CA, Le Marchand L, Yamamato J, Stram DO, Sheng X, Kolonel LN, et al. A common genetic risk factor for colorectal and prostate cancer. Nature genetics. 2007;39(8):954-6. Epub 2007/07/10. doi: 10.1038/ng2098. PubMed PMID: 17618282; PubMed Central PMCID: PMC2391283.

49. Hutter CM, Slattery ML, Duggan DJ, Muehling J, Curtin K, Hsu L, et al. Characterization of the association between 8q24 and colon cancer: gene-environment exploration and meta-analysis. BMC cancer. 2010;10:670. Epub 2010/12/07. doi: 10.1186/1471-2407-10-670. PubMed PMID: 21129217; PubMed Central PMCID: PMC3017062.

50. Kocarnik JD, Hutter CM, Slattery ML, Berndt SI, Hsu L, Duggan DJ, et al. Characterization of 9p24 risk locus and colorectal adenoma and cancer: gene-environment interaction and meta-analysis. Cancer epidemiology, biomarkers & prevention : a publication of the American Association for Cancer Research, cosponsored by the American Society of Preventive Oncology. 2010;19(12):3131-9. Epub 2010/10/28. doi: 10.1158/1055-9965.EPI-10-0878. PubMed PMID: 20978172; PubMed Central PMCID: PMC3005543.

51. Tenesa A, Farrington SM, Prendergast JG, Porteous ME, Walker M, Haq N, et al. Genome-wide association scan identifies a colorectal cancer susceptibility locus on 11q23 and replicates risk loci at 8q24 and 18q21. Nature genetics. 2008;40(5):631-7. Epub 2008/04/01. doi: 10.1038/ng.133. PubMed PMID: 18372901; PubMed Central PMCID: PMC2778004.

52. Tomlinson IP, Carvajal-Carmona LG, Dobbins SE, Tenesa A, Jones AM, Howarth K, et al. Multiple common susceptibility variants near BMP pathway loci GREM1, BMP4, and BMP2 explain part of the missing heritability of colorectal cancer. PLoS genetics. 2011;7(6):e1002105. Epub 2011/06/10. doi: 10.1371/journal.pgen.1002105. PubMed PMID: 21655089; PubMed Central PMCID: PMC3107194.

53. Houlston RS, Webb E, Broderick P, Pittman AM, Di Bernardo MC, Lubbe S, et al. Meta-analysis of genome-wide association data identifies four new susceptibility loci for colorectal cancer. Nature genetics. 2008;40(12):1426-35. Epub 2008/11/18. doi: 10.1038/ng.262. PubMed PMID: 19011631; PubMed Central PMCID: PMC2836775.

54. Jaeger E, Webb E, Howarth K, Carvajal-Carmona L, Rowan A, Broderick P, et al. Common genetic variants at the CRAC1 (HMPS) locus on chromosome 15q13.3 influence colorectal cancer risk. Nature genetics. 2008;40(1):26-8. Epub 2007/12/18. doi: 10.1038/ng.2007.41. PubMed PMID: 18084292.

55. Broderick P, Carvajal-Carmona L, Pittman AM, Webb E, Howarth K, Rowan A, et al. A genome-wide association study shows that common alleles of SMAD7 influence colorectal cancer risk. Nature genetics. 2007;39(11):1315-7. Epub 2007/10/16. doi: 10.1038/ng.2007.18. PubMed PMID: 17934461.

56. Peters U, Hutter CM, Hsu L, Schumacher FR, Conti DV, Carlson CS, et al. Meta-analysis of new genome-wide association studies of colorectal cancer risk. Human genetics. 2012;131(2):217-34. Epub 2011/07/16. doi: 10.1007/s00439-011-1055-0. PubMed PMID: 21761138; PubMed Central PMCID: PMC3257356.

57. Whiffin N, Hosking FJ, Farrington SM, Palles C, Dobbins SE, Zgaga L, et al. Identification of susceptibility loci for colorectal cancer in a genome-wide meta-analysis. Human molecular genetics. 2014. Epub 2014/04/17. doi: 10.1093/hmg/ddu177. PubMed PMID: 24737748.

58. Spain SL, Carvajal-Carmona LG, Howarth KM, Jones AM, Su Z, Cazier JB, et al. Refinement of the associations between risk of colorectal cancer and polymorphisms on chromosomes 1q41 and 12q13.13. Human molecular genetics. 2012;21(4):934-46. Epub 2011/11/15. doi: 10.1093/hmg/ddr523. PubMed PMID: 22076443; PubMed Central PMCID: PMC3263985.

59. Whiffin N, Dobbins SE, Hosking FJ, Palles C, Tenesa A, Wang Y, et al. Deciphering the genetic architecture of low-penetrance susceptibility to colorectal cancer. Human molecular genetics. 2013. Epub 2013/08/02. doi: 10.1093/hmg/ddt357. PubMed PMID: 23904454.

60. Wang H, Haiman CA, Burnett T, Fortini BK, Kolonel LN, Henderson BE, et al. Fine-mapping of Genome-wide Association Study-identified Risk Loci for Colorectal Cancer in African Americans. Human molecular genetics. 2013. Epub 2013/07/16. doi: 10.1093/hmg/ddt337. PubMed PMID: 23851122.

61. Carvajal-Carmona LG, Cazier JB, Jones AM, Howarth K, Broderick P, Pittman A, et al. Fine-mapping of colorectal cancer susceptibility loci at 8q23.3, 16q22.1 and 19q13.11: refinement of association signals and use of in silico analysis to suggest functional variation and unexpected candidate target genes. Human molecular genetics. 2011;20(14):2879-88. Epub 2011/05/03. doi: 10.1093/hmg/ddr190. PubMed PMID: 21531788; PubMed Central PMCID: PMC3118761.

62. Pittman AM, Naranjo S, Jalava SE, Twiss P, Ma Y, Olver B, et al. Allelic variation at the 8q23.3 colorectal cancer risk locus functions as a cis-acting regulator of EIF3H. PLoS genetics. 2010;6(9):e1001126. Epub 2010/09/24. doi: 10.1371/journal.pgen.1001126. PubMed PMID: 20862326; PubMed Central PMCID: PMC2940760.

63. Tuupanen S, Turunen M, Lehtonen R, Hallikas O, Vanharanta S, Kivioja T, et al. The common colorectal cancer predisposition SNP rs6983267 at chromosome 8q24 confers potential to enhanced Wnt signaling. Nature genetics. 2009;41(8):885-90. Epub 2009/06/30. doi: 10.1038/ng.406. PubMed PMID: 19561604.

64. Pittman AM, Webb E, Carvajal-Carmona L, Howarth K, Di Bernardo MC, Broderick P, et al. Refinement of the basis and impact of common 11q23.1 variation to the risk of developing colorectal cancer. Human molecular genetics. 2008;17(23):3720-7. Epub 2008/08/30. doi: 10.1093/hmg/ddn267. PubMed PMID: 18753146.

65. Pittman AM, Naranjo S, Webb E, Broderick P, Lips EH, van Wezel T, et al. The colorectal cancer risk at 18q21 is caused by a novel variant altering SMAD7 expression. Genome research. 2009;19(6):987-93. Epub 2009/04/28. doi: 10.1101/gr.092668.109. PubMed PMID: 19395656; PubMed Central PMCID: PMC2694486.

Table A. Overview of studies included in the present study population.

| Study Name | Abbreviation | Design | Country | Cases | Controls | Age range  (years) | Mean Age  (years) | Female  (%) | Covariates used in analysis |
| --- | --- | --- | --- | --- | --- | --- | --- | --- | --- |
|  |  |  |  |  |  |  |  |  |  |
| Association STudy Evaluating RISK for sporadic colorectal cancer | ASTERISK | case-control | France | 948 | 947 | 40-99 | 65.3 | 41.3 | age, gender, 3 PCs, batch |
| Colorectal Cancer Studies 2&3 | Colo2&3 | case-control | U.S. | 87 | 125 | 38-86 | 65.2 | 44.8 | age, gender, 3 PCs |
| Colon Cancer Family Registry^a^ | CCFR | case-control | U.S., Canada, Australia | 1,171 | 983 | 19-88 | 55.5 | 51.8 | age, gender, 3 PCs, center |
| Darmkrebs: Chancen der Verhütung durch Screening | DACHS | case-control | Germany | 2,376 | 2,206 | 33-98 | 68.7 | 39.9 | age, gender, PCs |
| Diet, Activity and Lifestyle Study | DALS | case-control | U.S. | 1,116 | 1,174 | 30-79 | 65.2 | 44.9 | age, gender, 3 PCs, center |
| Health Professionals Follow-up Study | HPFS | cohort | U.S. | 403 | 402 | 48-83 | 65.2 | 0 | age, 3PCs |
| Multiethnic Cohort Study | MEC | cohort | U.S. | 328 | 346 | 45-76 | 63.0 | 46.4 | age, gender, 3 PCs |
| Nurses’ Health Study | NHS | cohort | U.S. | 553 | 955 | 44-69 | 59.8 | 100 | age, 3 PCs |
| Ontario Familial Colorectal Cancer Registry | OFCCR | case-control | Canada | 650 | 522 | 31-79 | 64.1 | 52.0 | age, gender, 3 PCs |
| Physician’s Health Study | PHS | cohort | U.S. | 382 | 389 | 40-84 | 58.4 | 0 | age, 3 PCs, smoking |
| Postmenopausal Hormone study | PMH | case-control | U.S. | 280 | 122 | 50-75 | 64.8 | 100 | age, 3 PCs |
| Prostate, Lung, Colorectal and Ovarian Cancer Screening Trial | PLCO | cohort | U.S. | 1,019 | 2,391 | 55-75 | 64.0 | 30.8 | age, gender, 3 PCs, center |
| VITamins And Lifestyle | VITAL | cohort | U.S. | 285 | 288 | 50-76 | 66.5 | 47.6 | age, gender, 3 PCs |
| Women’s Health Initiative | WHI | cohort | U.S. | 1,476 | 2,538 | 50-79 | 67.4 | 100 | age, 3 PCs, region |
| Health Professionals Follow-up Study, Adenoma Set | HPFS Ad | cohort | U.S. | 313 | 345 | 48-81 | 60.7 | 0 | age, 3 PCs |
| Nurses’ Health Study, Adenoma Set | NHS Ad | cohort | U.S. | 513 | 578 | 44-69 | 57.0 | 100 | age, 3 PCs |

Abbreviations: PCs, principal components

^a^CCFR is a collaborating study with GECCOTable B. Association results in 11,900 cases and 14,311 controls for 31 risk variants previously reported in GWAS of CRC (index SNPs).

| **Locus** | **Index SNP** | **Level** ^a^ | **Genetic**  **region** | **# SNPs**  **in**  **region** | **Risk/ other allele** | **Risk allele freq** | **OR** ^b,c^ | **Low** | **Up** | ***P*** | ***P*-het** | **GWAS** ^d^ | **Reported OR in**  **GWAS** | **Studies that overlap with GECCO** |
| --- | --- | --- | --- | --- | --- | --- | --- | --- | --- | --- | --- | --- | --- | --- |
|  |  |  |  |  |  |  |  |  |  |  |  |  |  |  |
| 1q25.3 | rs10911251 | *** | *LAMC1* | 1886 | A/C | 0.54 | 1.10 | 1.06 | 1.15 | 3.0E-06 | 6.7E-01 | 1,17 | 1.09 | GECCO study |
| 1q41 | rs6687758 | * | *DUSP10/CICP13* | 1885 | G/A | 0.22 | 1.05 | 1.00 | 1.10 | 3.5E-02 | 1.3E-01 | 2 | 1.09 | CCFR |
|  | rs6691170 |  | *DUSP10/CICP13* | 2096 | T/G | 0.40 | 1.02 | 0.98 | 1.06 | 4.0E-01 | 7.1E-01 | 2 | 1.06 | CCFR |
| 2q32.3 | rs11903757 | *** | *NABP1/SDPR* | 1536 | C/T | 0.16 | 1.12 | 1.05 | 1.18 | 3.4E-04 | 1.5E-01 | 1 | 1.16 | GECCO study |
| 3q26.2 | rs10936599 |  | *MYNN* | 1651 | C/T | 0.75 | 0.99 | 0.95 | 1.04 | 7.0E-01 | 4.5E-01 | 2 | 1.08 | CCFR |
| 5q31.1 | rs647161 | ** | *PITX1/ H2AFY* | 1499 | A/C | 0.66 | 1.06 | 1.02 | 1.11 | 2.9E-03 | 2.5E-01 | 3,17 | 1.11 | Replicated in GECCO |
| 6p21 | rs1321311 |  | *SRSF3/CDKN1A* | 2364 | A/C | 0.20 | 1.04 | 1.00 | 1.09 | 5.9E-02 | 1.4E-02 | 4 | 1.10 |  |
| 8q23.3 | rs16892766 | *** | *TRPS1/EIF3H* | 1432 | C/A | 0.09 | 1.24 | 1.16 | 1.33 | 3.7E-10 | 7.5E-01 | 5 | 1.25 |  |
| 8q24 | rs6983267 | *** | *SRRM1P1/POU5F1B/MYC* | 2257 | G/T | 0.48 | 1.13 | 1.08 | 1.17 | 5.5E-10 | 7.6E-01 | 6-9 | 1.21 | OFCCR, MEC, COLO23, CCFR |
| 9p24 | rs719725 | *** | *TPD52L3/UHRF2/GLDC* | 1907 | A/C | 0.60 | 1.07 | 1.03 | 1.11 | 4.5E-04 | 7.9E-01 | 7,10 | 1.07 | OFCCR, CCFR |
| 10p14 | rs10795668 | * | *KRT8P16/TCEB1P3* | 2363 | G/A | 0.67 | 1.04 | 1.00 | 1.08 | 4.8E-02 | 7.8E-01 | 5 | 1.12 |  |
| 11q13.4 | rs3824999 | *** | *POLD3* | 1788 | G/T | 0.53 | 1.08 | 1.04 | 1.12 | 2.7E-05 | 6.3E-01 | 4 | 1.08 |  |
| 11q23 | rs3802842 | *** | *C11orf93* | 1830 | C/A | 0.27 | 1.12 | 1.07 | 1.17 | 9.2E-08 | 2.9E-01 | 11 | 1.11 | DACHS, OFCCR |
| 12p13.32 | rs10774214 | * | *RPL18P9/CCND2* | 1656 | T/C | 0.38 | 1.04 | 1.00 | 1.09 | 3.0E-02 | 5.7E-01 | 3,17 | 1.09 | Replicated in GECCO |
|  | rs3217810 | *** | *CCND2* | 1571 | T/C | 0.12 | 1.22 | 1.13 | 1.31 | 1.0E-07 | 4.7E-01 | 1,17 | 1.20 | GECCO study |
|  | rs3217901 | *** | *CCND2* | 1539 | G/A | 0.41 | 1.11 | 1.07 | 1.16 | 3.7E-07 | 7.0E-01 | 1 | 1.10 | GECCO study |
| 12q13.13 | rs11169552 | * | *DIP2B/ATF1* | 1310 | C/T | 0.75 | 1.05 | 1.01 | 1.09 | 2.4E-02 | 2.8E-01 | 2 | 1.09 | CCFR |
|  | rs7136702 | *** | *LARP4/DIP2B* | 967 | T/C | 0.33 | 1.09 | 1.04 | 1.14 | 7.1E-04 | 1.8E-01 | 2 | 1.06 | CCFR |
| 12q24.21 | rs59336 | *** | *TBX3* | 2072 | T/A | 0.47 | 1.09 | 1.05 | 1.13 | 1.2E-05 | 1.2E-01 | 1 | 1.09 | GECCO study |
| 14q22.2 | rs1957636 | *** | *BMP4/ATP5C1P1/CDKN3* | 1613 | T/C | 0.41 | 1.05 | 1.01 | 1.09 | 7.3E-03 | 5.8E-02 | 12 | 1.08 | CCFR |
|  | rs4444235 | *** | *BMP4/MIR5580* | 1659 | C/T | 0.48 | 1.09 | 1.05 | 1.13 | 8.3E-06 | 9.7E-01 | 12,13 | 1.09 | CCFR, DACHS, OFCCR |
| 15q13 | rs11632715 | ** | *SCG5/GREM1* | 1735 | A/G | 0.47 | 1.05 | 1.01 | 1.09 | 7.8E-03 | 8.2E-01 | 12 | 1.12 | CCFR |
|  | rs16969681 | ** | *SCG5/GREM1* | 1692 | T/C | 0.07 | 1.09 | 1.02 | 1.16 | 8.9E-03 | 4.0E-01 | 12 | 1.18 | CCFR |
|  | rs4779584 | *** | *SCG5/GREM1* | 1701 | T/C | 0.19 | 1.13 | 1.08 | 1.19 | 3.0E-07 | 4.5E-01 | 12,14 | 1.15 | CCFR |
| 16q22.1 | rs9929218 | ** | *CDH1* | 1867 | G/A | 0.71 | 1.06 | 1.01 | 1.10 | 8.4E-03 | 5.9E-01 | 13 | 1.10 | DACHS, OFCCR |
| 18q21 | rs4939827 | *** | *SMAD7* | 1931 | T/C | 0.53 | 1.12 | 1.08 | 1.17 | 6.5E-10 | 4.0E-01 | 11,15 | 1.20 | DACHS, OFCCR |
| 19q13.1 | rs10411210 | * | *RHPN2* | 2307 | C/T | 0.90 | 1.07 | 1.00 | 1.14 | 3.7E-02 | 3.7E-01 | 13 | 1.15 | DACHS, OFCCR |
| 20p12.3 | rs2423279 |  | *BMP2/HAO1* | 1901 | C/T | 0.26 | 1.04 | 1.00 | 1.09 | 6.5E-02 | 1.4E-01 | 3,17 | 1.10 | Replicated in GECCO |
|  | rs4813802 | *** | *FERMT1/BMP2* | 1825 | G/T | 0.34 | 1.10 | 1.06 | 1.15 | 6.6E-06 | 2.4E-01 | 12,16 | 1.09 | CCFR |
|  | rs961253 | *** | *FERMT1/BMP2* | 1990 | A/C | 0.37 | 1.08 | 1.04 | 1.13 | 2.6E-05 | 7.0E-01 | 13 | 1.12 | DACHS, OFCCR, CCFR |
| 20q13.33 | rs4925386 | ** | *LAMA5* | 2173 | C/T | 0.68 | 1.07 | 1.02 | 1.11 | 1.6E-03 | 1.7E-01 | 2,16 | 1.08 | CCFR, GECCO study |

Abbreviations: SNP, single nucleotide polymorphism; Freq, frequency; OR, odds ratio; Low, 95% CI lower bound; Up, 95% CI upper bound; *P*-het, *P* value for test of heterogeneity across studies

^a^Level of statistical evidence: *SNP with *P* ≤ 0.05; **SNP with *P ≤* 0.01; ***SNP with *P*≤0.001

^b^As appropriate, associations adjusted for age in years, sex, first 3 principal components, study center, batch (ASTERISK), and smoking (PHS)

^c^Estimate calculated using the log-additive genetic model for each additional risk allele

^d^Previous genome-wide association studies: 1) Peters et al. Gastroenterology, 2012. 2) Houlston et al. Nat Genet, 2010. 3) Jia et al. Nat Genet, 2012. 4) Dunlop et al. Nat Genet, 2012. 5) Tomlinson et al. Nat Genet, 2008. 6) Tomlinson et al. Nat Genet, 2007. 7) Zanke et al. Nat Genet, 2007. 8) Haiman et al. Nat Genet, 2007. 9) Hutter et al. BMC Cancer, 2010. 10) Kocarnik et al. CEBP, 2010. 11) Tenesa et al. Nat Genet, 2008. 12) Tomlinson et al. PLoS Genet, 2011. 13) COGENT. Nat Genet, 2008. 14) Jaeger et al. Nat Genet, 2008. 15) Broderick et al. Nat Genet, 2007. 16) Peters et al. Hum Genet, 2011. 17) Whiffin et al. *Hum Mol Genet*, 2014. [[1](#_ENREF_1), [14](#_ENREF_14), [43-57](#_ENREF_43)]

Table C. Association results for SNPs reported as top functional candidates in previous studies that fine-mapped GWAS-identified CRC risk variants.

| **Locus** | **GWAS**  **SNP(s)** | **Reported top**  **functional**  **candidate(s)** | **Level** ^a^ | **Position** ^b^ | **Ref/ other allele** | **Ref allele freq** | **OR ^c,d^** | **Low** | **Up** | ***P*** | ***P*-het** | **Ref** ^e^ | **SNPs in this study showing “moderate” or “strong” functional evidence** | | | |
| --- | --- | --- | --- | --- | --- | --- | --- | --- | --- | --- | --- | --- | --- | --- | --- | --- |
|  |  |  |  |  |  |  |  |  |  |  |  |  | **SNP** | **Position** | **Distance (kb)** ^f^ | ***r^2^*** ^g^ |
|  |  |  |  |  |  |  |  |  |  |  |  |  |  |  |  |  |
| 1q41 | rs6687758 | rs11118883 |  | 222061022 | G/A | 0.65 | 0.97 | 0.93 | 1.01 | 1.1E-01 | 5.9E-01 | 1,2 | None | NA | NA | NA |
|  | rs6691170 | rs7547751 |  | 222065709 | T/C | 0.54 | 0.99 | 0.96 | 1.03 | 7.0E-01 | 7.3E-01 | 3 |  |  |  |  |
| 8q23.3 | rs16892766 | rs11986063 | *** | 117640315 | C/T | 0.90 | 0.83 | 0.78 | 0.89 | 1.1E-08 | 5.6E-01 | 4 | rs11986063 | 117640315 | 0 | reported |
|  |  | rs7837208 | *** | 117797872 | G/A | 0.89 | 0.89 | 0.83 | 0.95 | 2.5E-04 | 1.3E-01 | 4 |  |  |  |  |
|  |  | rs16888589 | *** | 117635602 | A/G | 0.91 | 0.80 | 0.75 | 0.86 | 3.3E-10 | 7.1E-01 | 4,5 |  |  |  |  |
| 8q24 | rs6983267 | rs6983267 | *** | 128413305 | G/T | 0.48 | 1.13 | 1.08 | 1.17 | 5.5E-10 | 7.6E-01 | 6 | rs6983267 | 128413305 | 0 | reported |
| 11q23 | rs3802842 | rs3802842 | *** | 111171709 | A/C | 0.73 | 0.89 | 0.86 | 0.93 | 9.2E-08 | 2.9E-01 | 7 | rs7130173 | 111154072 | 3 | 1.00 |
|  |  | rs3087967 | *** | 111156836 | C/T | 0.73 | 0.89 | 0.85 | 0.93 | 3.0E-08 | 3.4E-01 | 7 |  |  |  |  |
|  |  | rs10891246 | *** | 111170540 | G/A | 0.73 | 0.89 | 0.86 | 0.93 | 1.3E-07 | 3.2E-01 | 7 |  |  |  |  |
|  |  | rs7105857 | *** | 111170744 | T/C | 0.70 | 0.90 | 0.86 | 0.94 | 4.8E-07 | 2.4E-01 | 7 |  |  |  |  |
| 12q13.13 | rs11169552 | rs7972465 | ** | 50546125 | T/G | 0.66 | 0.94 | 0.90 | 0.98 | 1.7E-03 | 4.8E-01 | 1 | rs12818741 | 51032415 | 486 | 0.63 |
|  | rs7136702 | rs706793 |  | 50467769 | G/A | 0.56 | 1.02 | 0.98 | 1.06 | 3.1E-01 | 9.1E-01 | 1 |  |  |  |  |
| 14q22.2 | rs1957636 | rs12432287 |  | 54572981 | G/A | 0.69 | 0.98 | 0.94 | 1.01 | 2.0E-01 | 2.0E-01 | 8 | rs10130587 | 54419110 | 154 | 0.06 |
|  | rs4444235 | rs728425 |  | 54583187 | C/T | 0.69 | 0.98 | 0.94 | 1.02 | 3.0E-01 | 1.9E-01 | 8 | rs35107139 | 54419106 | 154 | 0.03 |
|  |  | rs8011813 |  | 54661615 | C/T | 0.68 | 0.96 | 0.93 | 1.00 | 6.7E-02 | 4.8E-01 | 8 | rs2071047 | 54418411 | 155 | 0.02 |
|  |  |  |  |  |  |  |  |  |  |  |  |  | rs17563 | 54417522 | 155 | 0.02 |
|  |  |  |  |  |  |  |  |  |  |  |  |  | rs12893484 | 54414738 | 158 | 0.03 |
| 15q13 | rs11632715 | rs1406389 | *** | 33009478 | A/T | 0.78 | 0.87 | 0.83 | 0.91 | 2.4E-09 | 2.4E-01 | 2 | rs1406389 | 33009478 | 0 | reported |
|  | rs16969681 | rs11632715 | ** | 33004247 | A/G | 0.47 | 1.05 | 1.01 | 1.09 | 7.8E-03 | 8.2E-01 | 8 | rs2293582 | 33010412 | 0.9 | 0.94 |
|  | rs4779584 | rs16969681 | ** | 32993111 | C/T | 0.93 | 0.92 | 0.86 | 0.98 | 8.9E-03 | 4.0E-01 | 8 | rs2293581 | 33010736 | 1 | 1.00 |
| 16q22.1 | rs9929218 | rs35158985 | * | 68796746 | A/G | 0.70 | 1.05 | 1.01 | 1.09 | 1.9E-02 | 6.6E-01 | 4 | rs9929218 | 68820946 | 0.2 | 1.00 |
|  |  | rs58548890 |  | 68791694 | -- | -- | -- | -- | -- | -- | -- | 4 |  |  |  |  |
|  |  | rs13339591 | * | 68809273 | T/C | 0.71 | 1.05 | 1.01 | 1.10 | 1.1E-02 | 5.0E-01 | 4 |  |  |  |  |
|  |  | rs2961 | ** | 68818903 | T/C | 0.71 | 1.05 | 1.01 | 1.10 | 9.9E-03 | 5.6E-01 | 4 |  |  |  |  |
|  |  | rs9929239 | ** | 68821126 | C/T | 0.72 | 1.06 | 1.01 | 1.10 | 8.4E-03 | 6.2E-01 | 4 |  |  |  |  |
|  |  | rs2059254 | * | 68817439 | C/T | 0.71 | 1.05 | 1.01 | 1.10 | 1.2E-02 | 5.1E-01 | 4 |  |  |  |  |
| 18q21 | rs4939827 | rs4939567 | *** | 46451873 | G/A | 0.55 | 1.13 | 1.09 | 1.18 | 1.6E-10 | 2.2E-01 | 2 | rs4939567 | 46451873 | 0 | reported |
|  |  | rs12953717 | *** | 46453929 | C/T | 0.56 | 0.90 | 0.87 | 0.93 | 5.5E-08 | 1.2E-01 | 9,10 | rs34007497 | 46451073 | 0 | reported |
|  |  | rs4464148 | *** | 46459032 | T/C | 0.66 | 0.93 | 0.89 | 0.97 | 8.3E-04 | 5.0E-01 | 9 | rs11874392 | 46453156 | 0.8 | 1.00 |
|  |  | rs58920878 | *** | 46449565 | C/G | 0.56 | 0.90 | 0.87 | 0.94 | 4.0E-07 | 2.9E-01 | 10 |  |  |  |  |
|  |  | rs8085824 | *** | 46449111 | T/C | 0.56 | 0.90 | 0.87 | 0.94 | 1.7E-07 | 1.7E-01 | 10 |  |  |  |  |
|  |  | rs34007497 | *** | 46451073 | C/G | 0.56 | 0.90 | 0.87 | 0.94 | 8.5E-08 | 1.8E-01 | 10 |  |  |  |  |
|  |  | rs4044177 |  | 46452701 | -- | -- | -- | -- | -- | -- | -- | 10 |  |  |  |  |
| 19q13.1 | rs10411210 | rs79812655 |  | 33517923 | -- | -- | -- | -- | -- | -- | -- | 4 | rs10411210 | 33532300 | 0 | reported |
|  |  | rs10411210 | * | 33532300 | C/T | 0.90 | 1.07 | 1.00 | 1.14 | 3.7E-02 | 3.7E-01 | 4 |  |  |  |  |
|  |  | rs28626308 |  | 33517515 | C/T | 0.94 | 1.09 | 0.96 | 1.25 | 1.8E-01 | 4.8E-02 | 4 |  |  |  |  |
|  |  | rs17841839 |  | 33517201 | C/T | 0.93 | 1.10 | 0.97 | 1.26 | 1.3E-01 | 4.2E-02 | 4 |  |  |  |  |
|  |  | rs73039426 | * | 33520961 | C/T | 0.93 | 1.14 | 1.03 | 1.27 | 1.4E-02 | 1.8E-01 | 4 |  |  |  |  |
|  |  | rs73039428 | * | 33521150 | A/G | 0.93 | 1.14 | 1.03 | 1.27 | 1.4E-02 | 1.9E-01 | 4 |  |  |  |  |
| 20q13.33 | rs4925386 | rs1741640 | *** | 60932414 | C/T | 0.77 | 1.11 | 1.05 | 1.19 | 6.9E-04 | 3.3E-01 | 2 | rs1741634 | 60925979 | 6 | 0.59 |
|  |  | rs2236202 | * | 60985164 | C/T | 0.75 | 1.08 | 1.02 | 1.14 | 1.4E-02 | 6.9E-01 | 2 | rs3810550 | 60912117 | 20 | 0.60 |

Abbreviations: SNP, single nucleotide polymorphism; Ref, reference; Freq, frequency; OR, odds ratio; Low, 95% CI lower bound; Up, 95% CI upper bound; *P*-het, *P* value for test of heterogeneity across studies

-- SNP did not pass quality control filters in present study

^a^Level of statistical evidence: *SNP with *P* ≤ 0.05; **SNP with *P ≤* 0.01; ***SNP with *P*≤0.001

^b^Based on NCBI build 37 data

^c^Adjusted for age in years, sex, first 3 principal components, study center, batch (ASTERISK only), and smoking (PHS only)

^d^Estimate calculated using the log-additive genetic model for each additional reference allele

^e^Previous fine-mapping studies: 1) Spain et al. Hum Mol Genet, 2012. 2) Whiffin et al. Hum Mol Genet, 2013. 3) Wang et al. Hum Mol Genet, 2013. 4) Carvajal-Carmona et al. Hum Mol Genet, 2011. 5) Pittman et al. PLoS Genet, 2010. 6) Tuupanen et al. Nat Genet, 2009. 7) Pittman et al. Hum Mol Genet, 2008. 8) Tomlinson et al. PLoS Genet, 2011. 9) Broderick et al. Nat Genet, 2007. 10) Pittman et al. Genome Res, 2009. [[52](#_ENREF_52), [55](#_ENREF_55), [58-65](#_ENREF_58)]

^f^Kilobases to nearest reported functional candidate

^g^Highest ***r^2^*** with reported functional candidate

Figure A. Regional association plots of CRC association signals for the 500-kb regions harboring the index SNPs 8q24/rs6983267 and 11q23/rs3802842. Each dot reflects the –log10 *P*-value of one SNP in the region. The purple diamond indicates the index SNP. The color of the other dots reflects correlation (*r^2^*) with the index SNP based on the 1000 Genomes Project European populations. Axes: left Y-axis shows –log10 of *P* values; x-axis shows SNP genomic position based on NCBI build 37; and right Y-axis shows the estimated recombination rate from the 1000 Genomes Project European populations.

1. 8q24/rs6983267


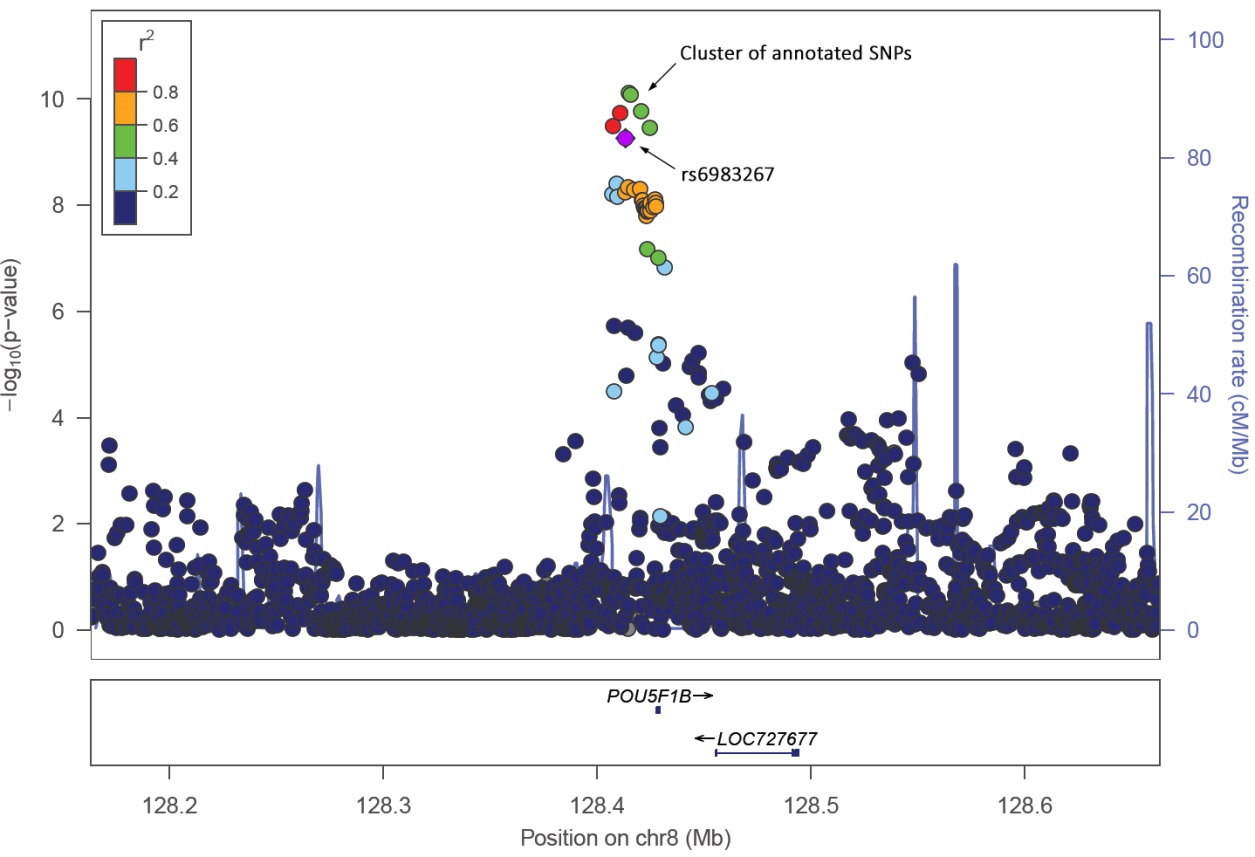


1. 11q23/rs3802842


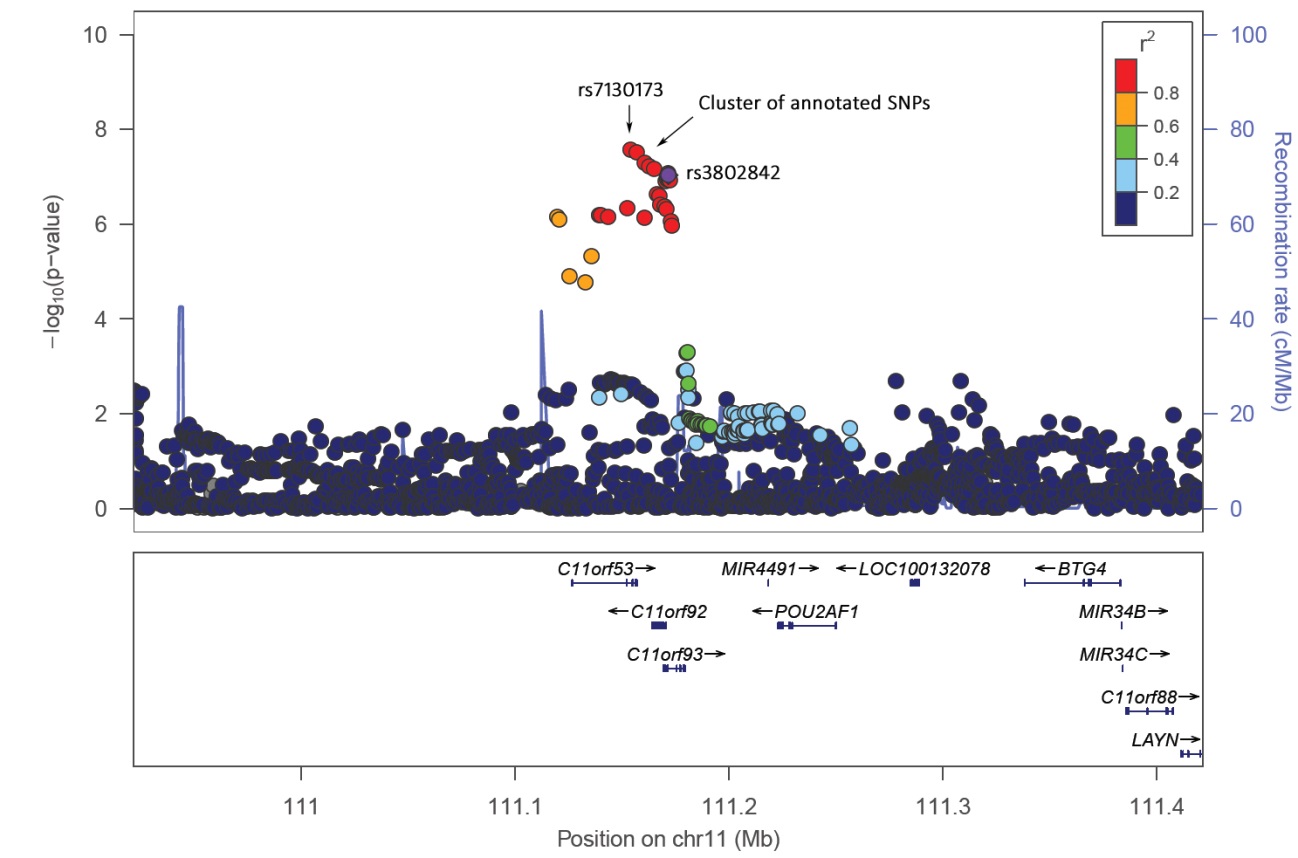


Figure B. ENCODE integrated regulation tracks for functional candidates in *GREM1*-15q13 (rs2293582, rs2293581, rs1406389)*.* A) Relative positions of the GWAS index SNP, rs4779584 (shown in red), the SNP showing the smallest *P*-value in the region, rs2293582, and two other SNPs highly correlated with the index SNP. Alignment of these SNPs with the UCSC Genes track showed these variants were located in the 5’ region of *GREM1* and may disrupt promoter activity. B) Focusing in on the boxed region in part A, the remaining tracks are a functional annotation of chr15:33,008,676-33,011,455 near the *GREM1* promoter region. C) Evidence of histone modifications associated with promoter activity. In cancerous cell lines, including the CRC CACO2 cells assayed by ENCODE, the signal was stronger than in normal colon muscle and mucosa tissue assayed by Roadmap. D) Strong signal for open chromatin structure associated with regulatory elements across various ENCODE cell lines. E) These variants were located in the peak of a bound protein. While rs2293582 was in a region that bound RNA Polymerase 2 transcription factor, the other two variants were in regions that bound SUZ12, a component of the repressive polycomb complex and may be involved in chromatin silencing.


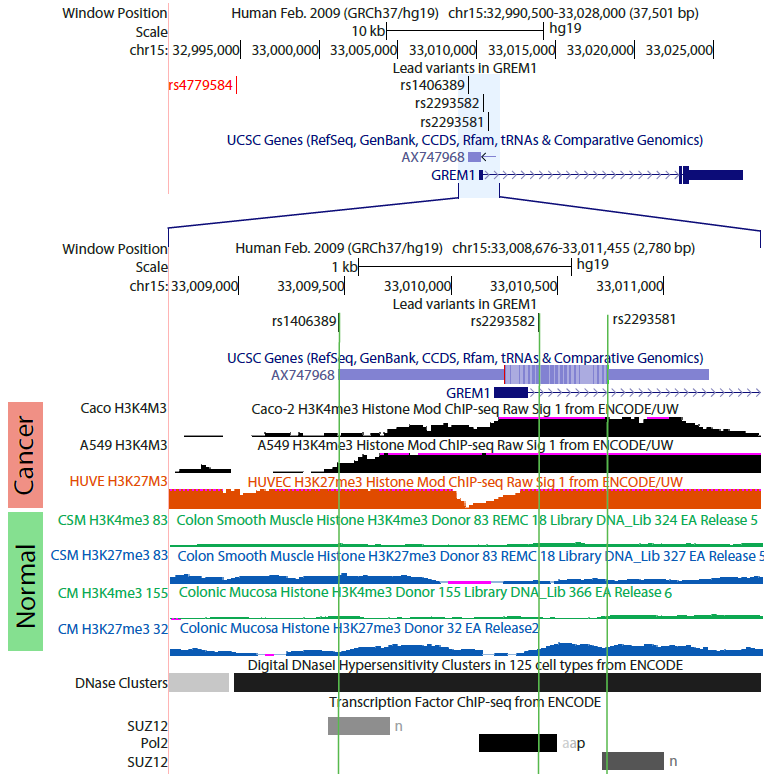


E)

D)

C)

B)

A)

Figure C. ENCODE integrated regulation tracks for functional candidates in *LAMA5*-20q13.33 (rs1741634, rs3810550)*.* A) Relative positions of the GWAS index SNP, rs4925386 (shown in red), and the SNP showing the smallest *P*-value in the region, rs1741634. Alignment of these SNPs with the UCSC Genes track showed these alleles were located in the intronic region of *LAMA5*. The remaining tracks are a functional annotation of the chr20:60,925,000-60,920,000 B) Evidence of open chromatin structure associated with regulatory elements across various ENCODE cell lines. The signal was stronger for the putative functional SNP than for the index SNP. C) Weak signal for GR transcription factor binding in the region containing rs1741634. D) Histone modifications associated with enhancer activity. The signal was stronger in cancerous cell lines, including the CRC HCT116 cells assayed by ENCODE, than in normal tissues (colon, rectal smooth muscle, colon mucosa) assayed by Roadmap. This suggested that rs1741634 may disrupt a putative enhancer element.


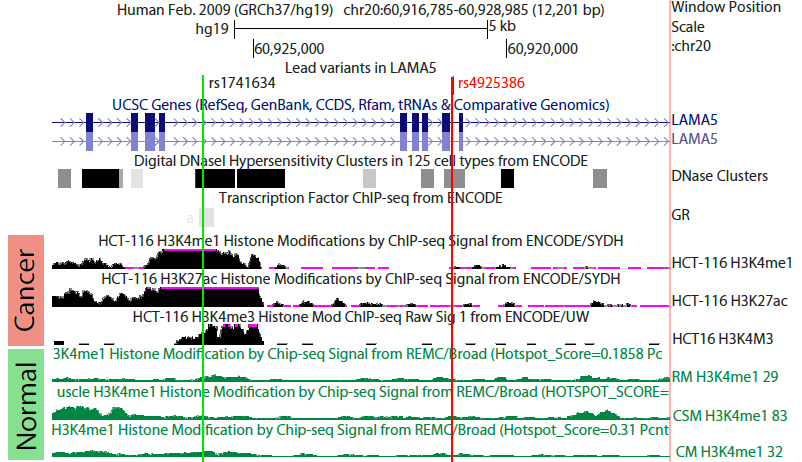


D)

C)

B)

A)

Figure D. Statistical power and minimum detectable odds ratios for SNPs with minor allele frequencies (MAFs) between 0.005 and 0.20. Statistical power estimated assuming 2-sided α=2.8E-05 (average Bonferroni-corrected α across 31 regions), imputation Rsq=0.9, and log-additive genetic effects.
